# Photocrystallographic and spectroscopic studies of a model (N,N,O)-donor square-planar nickel(II) nitro complex: in search of high-conversion and stable photoswitchable materials

**DOI:** 10.1107/S205225252001307X

**Published:** 2020-10-29

**Authors:** Sylwia E. Kutniewska, Adam Krówczyński, Radosław Kamiński, Katarzyna N. Jarzembska, Sébastien Pillet, Emmanuel Wenger, Dominik Schaniel

**Affiliations:** a Department of Chemistry, University of Warsaw, Żwirki i Wigury 101, Warsaw 02-089, Poland; b Université de Lorraine, CNRS, CRM2, F-54000 Nancy, France

**Keywords:** nitro group (photo)isomerization, photoswitchable materials, photocrystallography, IR spectroscopy, nickel complexes

## Abstract

A new, cheap, easy-to-synthesize and air-stable photoswitchable nickel(II) complex, QTNiNO_2_, is reported. The metal centre in QTNiNO_2_ is coordinated by a nitro group and a [2-methyl-8-amino­quinoline]-1-tetralone ligand. The compound crystallizes in the tetragonal space group *I*4_1_/*a* with one complex molecule comprising the asymmetric unit, and the crystals are stable under ambient conditions. Irradiation of the solid-state form of QTNiNO_2_ with 530–660 nm LED light at 160 K converts the ambidentate nitro moiety fully to the nitrito linkage isomer which is stable up to around 230 K, as indicated by IR spectroscopy measurements. The structures of all species present in the examined crystals and their thermal stability were confirmed via X-ray multi-temperature and photocrystallographic experiments. The impact of temperature on the (photo)isomerization reaction taking place in a single crystal was additionally investigated. The experimental results are supported by computational analyses of crystal packing and intermolecular interactions that influence the isomerization process studied.

## Abstract 2

A new, cheap, easy-to-synthesize and air-stable photoswitchable nickel nitro complex, in which the central metal atom is additionally chelated by a [2-methyl-8-amino­quinoline]-1-tetralone ligand, is reported. The compound was shown to undergo full light-induced isomerization from the *nitro* to *nitrito* form by irradiation with 530–660 nm LED light at 160 K, whereas the generated linkage isomer exhibits thermal stability up to around 230 K.

## 1. Introduction   

Chemical compounds exhibiting specific photoactive properties, either in solution or even more importantly in the solid state, have already found versatile applications in solar-energy conversion and other fields ranging from molecular electronics and photocatalysts through light-emitting devices (LEDs) to biolabels (Alibabaei *et al.*, 2013 ▸; Kamtekar *et al.*, 2010 ▸; Kong *et al.*, 2017 ▸; Reineke *et al.*, 2009 ▸). Photoswitchable transition-metal complexes, in which the metal centre (*e.g.* Ni, Co, Pd, Rh) is coordinated by ambidentate ligands, such as NO, NO_2_ or SO_2_ moieties, are the most representative examples of such systems (Kovalevsky *et al.*, 2005 ▸; Hatcher *et al.*, 2011 ▸, Hatcher, Christensen *et al.*, 2014 ▸; Cole *et al.*, 2018 ▸; Sylvester *et al.*, 2014 ▸; Bowes *et al.*, 2006 ▸; Schaniel *et al.*, 2019 ▸, 2018 ▸; Schaniel, Nicoul & Woike, 2010 ▸; Hatcher & Raithby, 2014 ▸, 2013 ▸; Cormary *et al.*, 2012 ▸; Coppens *et al.*, 2002 ▸). Under certain conditions they can be switched from the ground-state form to a metastable linkage isomer by light of a suitable wavelength, which may further influence their macroscopic properties, *e.g.* colour (photochromism) (Schaniel *et al.*, 2002 ▸; Mikhailov *et al.*, 2019 ▸) or refractive index (Goulkov *et al.*, 2010 ▸; Schaniel *et al.*, 2007 ▸). Efficient and industrially desirable photoswitchable materials should fulfil the following requirements: sufficient stability (*i.e.* the compound itself and its excited/metastable-state form should be chemically and thermodynamically stable at operating conditions), low cost, non-toxicity, and easy, high-yield synthesis, whereas the photo-induced process should be characterized by high conversion, controllability and reproducibility.

As far as nitro transition-metal coordination compounds are concerned, most of the reported systems are characterized by low- or medium-level conversion percentages to metastable linkage isomers (Fomitchev *et al.*, 1998 ▸; Schaniel *et al.*, 2010 ▸; Kovalevsky *et al.*, 2005 ▸). The first system of this kind, [Ni(dppe)(NO_2_)Cl] [dppe = 1,2-bis­(di­phenyl­phosphino)­ethane], which was confirmed to undergo 100% conversion between its ground state and the nitrito isomer, was described in the work by Warren *et al.* (2009 ▸). Raithby and co-workers later published a few more articles on transition-metal nitro-coordination compounds capable of achieving high-conversion levels upon light irradiation (Skelton *et al.*, 2015 ▸; Warren *et al.*, 2014 ▸; Hatcher & Raithby, 2014 ▸, 2013 ▸; Hatcher, Christensen *et al.*, 2014 ▸; Hatcher, Bigos *et al.*, 2014 ▸; Hatcher *et al.*, 2011 ▸, 2019 ▸). Nevertheless, up to now the metastable species have usually been present in the crystal structures only at relatively low temperatures.

Hence, in the current contribution, which details a part of our wider project dedicated to the search for novel functional materials (Kutniewska *et al.*, 2019 ▸; Jarzembska *et al.*, 2017 ▸), a new photo-active nitro-group nickel(II) complex is introduced. The compound designed and synthesized by us, QTNiNO_2_ (shown in the scheme[Chem scheme1] below), aside from the key nitro group, contains a [2-methyl-8-amino­quinoline]-1-tetralone (QT) ligand chelating the metal centre. The crystals are stable under standard conditions and exhibit promising photoswitchable properties. The nitro fragment photoisomerization in the solid state was studied in depth spectroscopically, (photo)crystallographically (including multi-temperature experiments) and also computationally. The reported compound opens up a whole group of interesting photoswitchable complexes to be investigated in the nearest future.
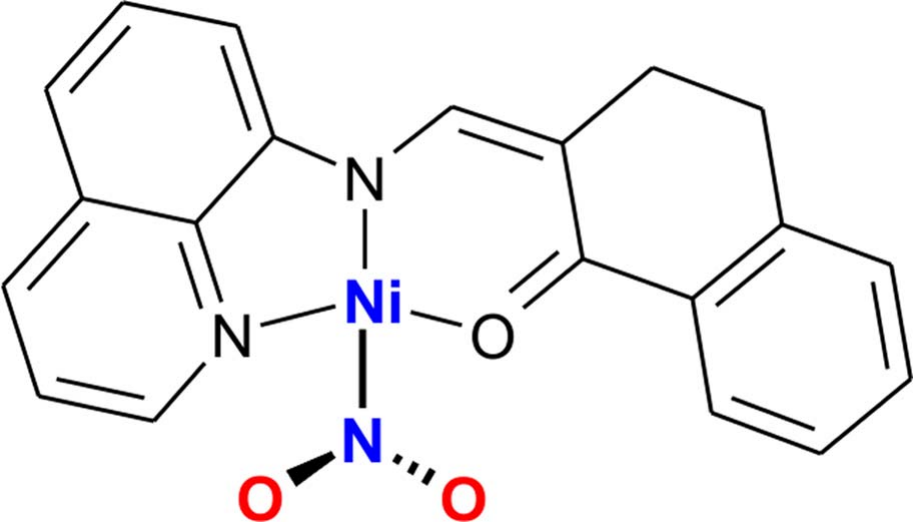


## 2. Experimental   

### 2.1. Synthesis   

The intermediate product essential for the preparation of QTNiNO_2_ was prepared according to the procedure in the literature (Dabrowski & Krówczyński, 1977 ▸). A mixture of 1.0 mmol 1-tetralone in 2.0 mmol ethyl formate was added to 1.3 mmol of molecular sodium in 20 ml Et_2_O and stirred for 12 h. After solvent evaporation the hy­droxy­methyl­eneketone sodium salt obtained was dissolved in 30 ml MeOH, then 1.0 mmol 8-amino­quinoline in 10 ml AcOH was added, and finally the mixture was neutralized with AcOH. The intermediate product obtained was not isolated, whereas its solution was brought to boil followed by the addition of 1.2 mmol Ni(OAc)_2_ in 10 ml MeOH. Next, heating of the solution was stopped while stirring was continued for the following 2 h. The dark-brown mixture was purified by filtration and *ca* 0.5 mmol LiNO_2_ in MeOH was added. The final product was cooled with an ice bath and filtered. The overall yield amounted to about 60%. Small brownish crystals suitable for single-crystal X-ray diffraction experiments were grown via the diffusion-crystallization method using *n*-hexane and MeOH as solvents. For more details see the supporting information (Whitaker *et al.*, 1995 ▸).

### 2.2. X-ray diffraction   

All X-ray diffraction datasets were collected using a Rigaku Oxford Diffraction SuperNova single-crystal diffractometer equipped with a CCD-type area detector and copper microfocus X-ray source. Additionally, our homemade light-delivery device (Kamiński *et al.*, 2016 ▸), specifically redesigned to fit the SuperNova setup to facilitate *in situ* photocrystallographic experiments, was installed. The data-collection strategy employed the mounted light-delivery device and was prepared using the native diffractometer software (Rigaku Oxford Diffraction, 2020 ▸). All data collections were performed in complete darkness. The overall experimental procedure was as follows: crystal mounting at room temperature and cooling to the desired temperature, measurement of a ground-state structure and/or irradiation of the crystal for ∼3 h (during this time the crystal was continuously rotated to ensure uniform exposure), measurements of the crystal at various temperatures ranging from 100 to 240 K (see text) in 20 K steps. Further data processing was consistent for all data collections. All crystal structures were solved using an intrinsic phasing method as implemented in the *SHELXT* program (Sheldrick, 2015 ▸), and refined with the *JANA* package (Petříček *et al.*, 2014 ▸) within the independent atom model approximation. For more details see the supporting information (Allen, 2002 ▸; Groom *et al.*, 2016 ▸; Fournier & Coppens, 2014 ▸; Schmøkel *et al.*, 2010 ▸). The CIFs are provided in the supporting information, but can also be retrieved from the Cambridge Structural Database (CCDC deposition numbers: 1975196-202, 1975732-736).

### 2.3. IR spectroscopy   

All IR measurements were performed using the Nicolet 5700 FT-IR spectrometer equipped with a closed-cycle cryostat. The sample was ground, mixed with spectroscopic grade KBr, pressed into pellets and glued to the cold-finger of the cryostat using silver-paste thermal adhesive. During measurements the sample was kept under vacuum inside the cryostat. Irradiation of the sample was achieved through the cryostat window using various LEDs (Thorlabs L and LP series), central wavelengths of which covered the range from violet to red (from 385 to 735 nm).

### 2.4. Theoretical computations   

All computations were performed with the *GAUSSIAN16* package (Frisch *et al.*, 2016 ▸). In the case of quantum-mechanics/molecular-mechanics (QM/MM) computations, the model of a ground-state or metastable-state molecule in a crystal environment (Kamiński *et al.*, 2010 ▸) is composed of a central molecule (QM part) and a shell (MM part) cut-out from the studied crystal structure with a radius of 12 Å [all C—H distances were set to the neutron-normalized values (Allen & Bruno, 2010 ▸; Allen *et al.*, 1987 ▸)]. The density functional level of theory (DFT(B3LYP)/6-311++G** (Becke, 1988 ▸; Perdew, 1986 ▸; Lee *et al.*, 1988 ▸; Krishnan *et al.*, 1980 ▸; Clark *et al.*, 1983 ▸; McLean & Chandler, 1980 ▸) was applied for the optimization of the central molecule and calculation of Hirshfeld atomic charges (Hirshfeld, 1977 ▸) used later for the purpose of molecular shell approximation with the Universal Force Field (UFF) (Rappé *et al.*, 1992 ▸). Dimer interaction energies, isolated-molecule geometry optimizations and normal-mode frequencies were also calculated at the DFT(B3LYP)/6-311++G** level of theory. In the case of interaction energy computations, the Grimme empirical dispersion correction (Grimme, 2006 ▸, 2004 ▸) modified by the Becke–Johnson damping function (Grimme *et al.*, 2010 ▸, 2011 ▸), and correction for BSSE (Boys & Bernardi, 1970 ▸; Simon *et al.*, 1996 ▸) were applied. The input files were generated using the *CLUSTERGEN* program (Kamiński *et al.*, 2013 ▸).

## 3. Results and discussion   

### 3.1. Crystal structure and its temperature evolution   

**Figure 1:**
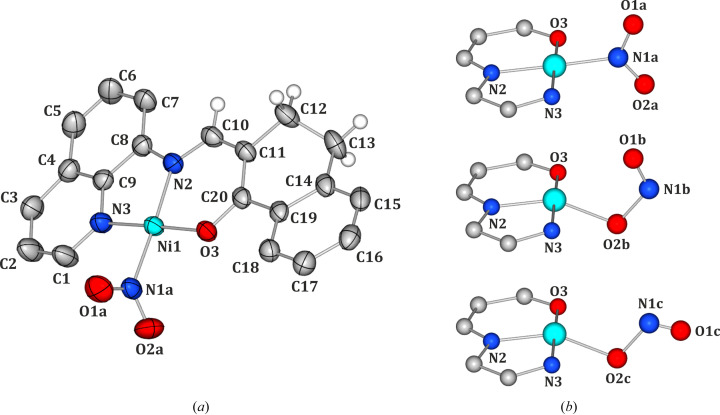
Molecular structure of the QTNiNO_2_ complex present in the non-irradiated 180 K crystal structure (selected hydrogen atoms have been omitted for clarity). (*a*) Ground state shown of the nitro η^1^-N(O)_2_ form (atomic thermal motion is represented as ellipsoids at the 50% probability level). (*b*) All NO_2_-group isomers disentangled (only the Ni atom environment is shown for clarity); from the top: nitro, *endo*-nitrito η^1^-ONO and *exo*-nitrito η^1^-(O)_2_N.

The crystal structure of the synthesized and crystallized QTNiNO_2_ complex was first examined at 100 K. It appears that the compound crystallizes in the tetragonal space group *I*4_1_/*a*, with one molecule in the asymmetric unit (Fig. 1 ▸). The QT ligand coordinates to the nickel centre via all the electron-donating atoms, *i.e.* the quinolione N3 and bridging N2 nitro­gen atoms, and the O3 oxygen atom from the carbonyl group. Overall, the organic (N,N,O)-ligand moiety is approximately flat. The angle between the planes of the quinoline ring and the aromatic six-membered ring of the tetralone fragment amounts to *ca* 17°. Quite interestingly, at 100 K in the absence of light, some disorder of the nitro group is already observed. The nitro binding mode η^1^-N(O)_2_ dominates in the crystal structure, while the moiety is oriented approximately perpendicularly to the plane of the organic ligand, whereas the nickel centre adopts a square coordination. In addition, a tiny fraction (*ca* 2%) of the *endo*-nitrito η^1^-ONO isomer is present. The most relevant compound and crystal parameters, data collection and refinement details are summarized in Tables 1 ▸ and S1 of the supporting information.

**Figure 2:**
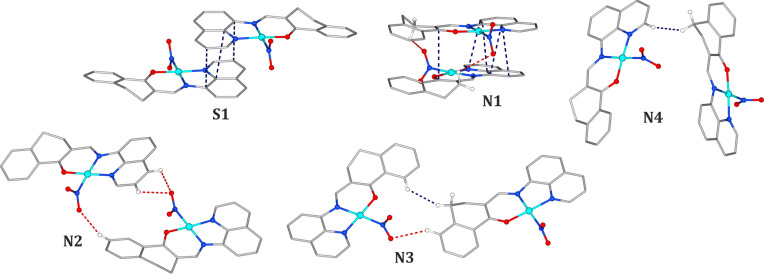
The main structural motifs present in the QTNiNO_2_ structure (only the nitro isomer is considered: **S** – π-stacking-based motifs, **N** – motifs supported by nitro group interactions).

Overall, the analysed crystal structure is rather compact and its relatively high symmetry is manifested by the molecular arrangement in space, whereby the organic and nitro ligands are grouped separately (Fig. S1 in the supporting information). This leads to a set of structural motifs, out of which the most relevant are depicted in Fig. 2 ▸, and their geometrical and energetic features are presented in Table 2 ▸ (taking into account the nitro isomer only). The crystal structure of QTNiNO_2_ is stabilized by various weak interactions, such as C−H⋯π, π-stacking, as well as C−H⋯O hydrogen-bond-like contacts involving the nitro ligand. For instance, notable π-stacking interactions are formed between the quinoline rings (the distance between ring planes is equal to *ca* 3.55 Å) and link together the complex molecules in the **S1** motif. The total interaction energy of **S1** amounts to about −84.4 kJ mol^−1^. Nevertheless, as far as the photo-isomerization reaction is concerned, the nitro group interactions in the crystal structure are the most essential. In this respect, the main structural motif identified in the crystal structure involves two molecules related by the 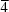 inversion axis (motif **N1**). In this motif the molecules interact via π⋯π stacking contacts between the organic ligands (namely two quinolone fragments interact with each other as do the tetralone aromatic rings) as well as, more importantly, via C12−H12a⋯O1a interactions between the tetralone CH_2_ fragment groups and nitro ligands. This is the most energetically stable dimer of all, engaging the nitro group (−127.9 kJ mol^−1^). Other significant interactions employing the nitro group are also realized via C−H⋯O interactions and stabilize the **N2** and **N3** motifs (*e.g.* C17−H17⋯O1a or C17−H17⋯O1a, and C15−H15⋯O1a contacts). Their total interaction energies amount to about −43.8 and −17.2 kJ mol^−1^, respectively. Finally, a less obvious **N4** dimer is encountered. In this motif one molecule somewhat encapsulates the nitro group of the second moiety, while the two species mostly interact via weak dispersive interactions including short H⋯H contacts (namely H1⋯H13a), which yield a total interaction energy of −19.9 kJ mol^−1^.

**Figure 3:**
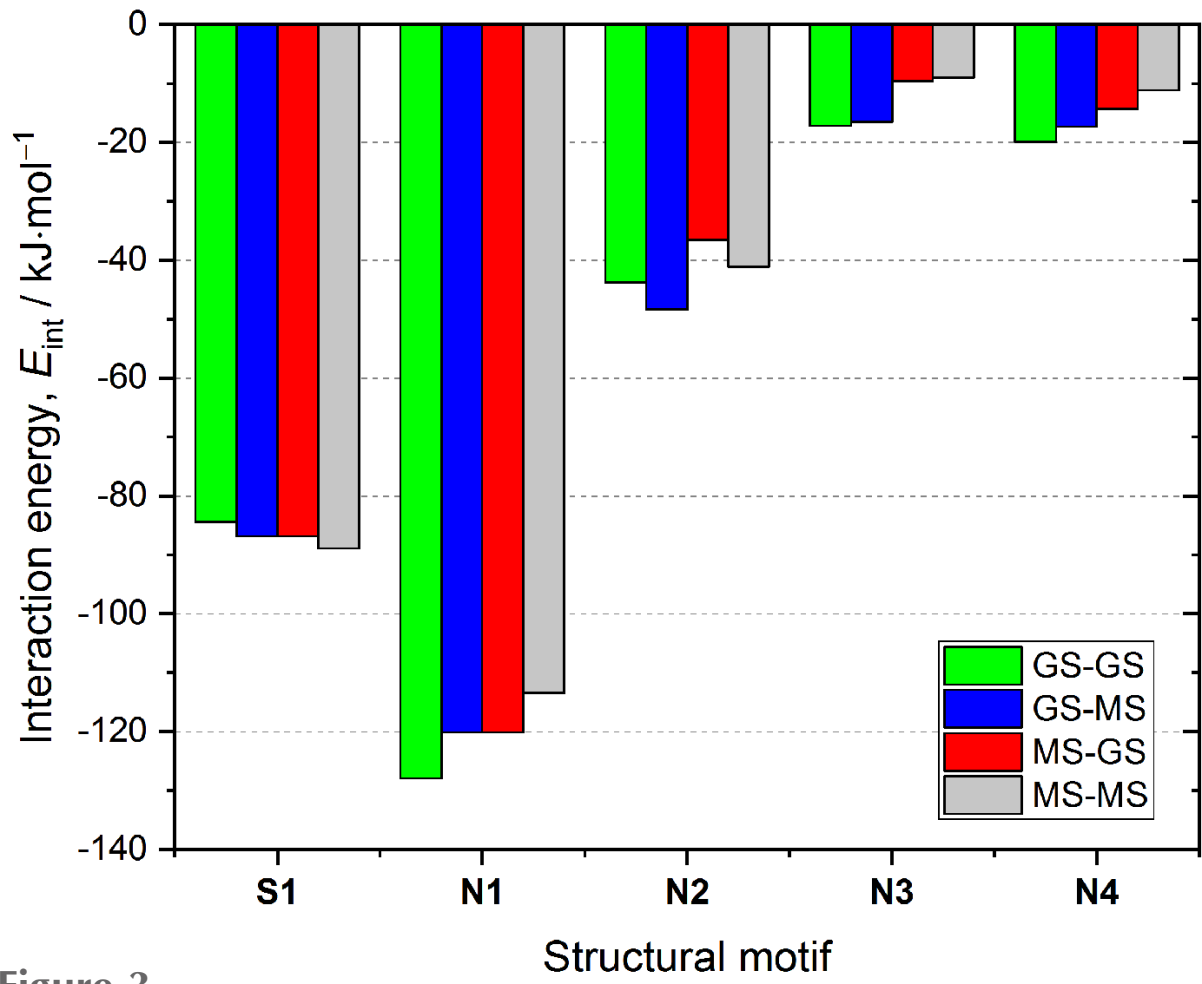
Motif interaction energies for dimers containing one or two molecules in the *endo*-nitrito metastable form, compared with the ground-state dimers (Table 2 ▸). The crystal structure at 100 K was used for all computations. For numerical data see the supporting information. GS-MS denotes that the first molecule is in the ground state and the second molecule is in the excited state; the first molecule is always the left one as shown in Fig. 2 ▸ (except for **S1** and **N2**, which are symmetric motifs).

Since the NO_2_ ligand is disordered, it is particularly interesting to see how much the interaction pattern of a single molecule, and thus its stabilization energy in the crystal structure, changes when the nitro group is substituted by the isomeric *endo*-nitrito binding mode. The interaction energy values describing the motifs from Table 2 ▸, in which the nitro form transformed into its *endo*-nitrito isomer, are presented in Fig. 3 ▸. Generally, all **N**-type motifs become less advantageous in terms of the interaction energy when the nitro group undergoes switching. The largest absolute change (+14.5 kJ mol^−1^) is observed for the most energetically stable motif **N1** which constitutes only about 11% of the total energy of this dimer. In this case the O1 oxygen atom, involved in the interaction with the organic ligand, occupies the same position. Only the O2 and N1 atoms change their locations. As a result the intermolecular interaction pattern remains essentially the same, from which we can conclude that a different distribution of electron density in the metastable state is most likely responsible for the observed weakening of the interaction. For other motifs the absolute interaction energy change is smaller, however, the relative changes are generally more significant (for example *ca* 44% for **N4**). Among the motifs engaging the ONO group, the behaviour of the **N2** dimer is most interesting. When the nitro group involved in the C2/3−H2/3⋯O2a interaction is switched to the *endo*-nitrito form, this motif becomes more stable. This is due to the fact that, along with the H2/3⋯O2a contacts becoming more distant, an extra C2−H2⋯N1b interaction is formed. However, an opposite trend is observed for the second nitro group in this motif. Consequently, the overall effect when both groups are switched to the metastable-state form is that the motif becomes less energetically stable when compared with the situation when both molecules constitute the nitro isomers. In turn, the increasing energetic stability of the **S1** motif upon isomerization can be explained similarly as above by the formation of a new interaction which occurs between the O2 atom and the quinolone moiety of the neighbouring molecule.

Finally, in view of the further photoactivity studies of the QTNiNO_2_ complex, it is worth looking at the amount of space that the nitro group can utilize during the photo-reaction. The reaction cavity volume computed for the 100 K crystal structure [using *MERCURY* (Macrae *et al.*, 2020 ▸); the rolling-probe method: probe radius of 1.2 Å, grid spacing of 0.1 Å] is equal to 33.2 Å^3^ per one complex molecule. This volume is compar­able to the respective literature-reported values derived for other NO_2_ metal complexes undergoing photo-induced transformations (Hatcher, Bigos *et al.*, 2014 ▸; Hatcher & Raithby, 2017 ▸).

**Figure 4:**
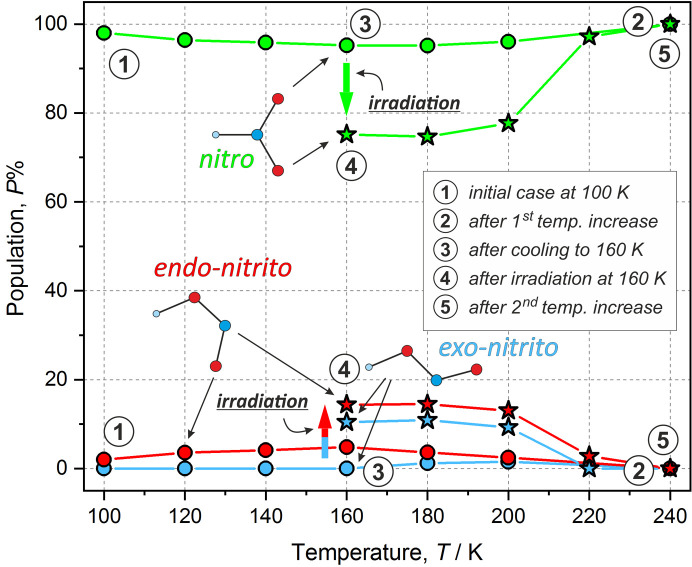
Populations of all states *versus * temperature (including irradiated crystal structures): the crystal was first temperature-scanned from 100 to 240 K, cooled down to 160 K and irradiated, and then the temperature was increased again. Green solid lines – nitro isomer, red – *endo*-nitrito form, blue – *exo*-nitrito form; circles denote the first temperature scan, stars denote the second scan (after irradiation); numbers in the circles denote important stages of the experiment; error bars are smaller than the symbols. For numerical data see the supporting information.

The presence of two linkage isomers in the QTNiNO_2_ crystal structure at 100 K suggests that the nitro-to-nitrito transformation triggered by temperature rise and/or photoexcitation could be expected. There is already a number of examples in the literature of nickel nitro coordination compounds exhibiting such behaviour in the solid-state upon either photo or thermal activation (Chattopadhyay *et al.*, 2005 ▸; Nakamoto *et al.*, 1958 ▸; Finney *et al.*, 1981 ▸; Kutniewska *et al.*, 2019 ▸). Consequently, to verify these anticipations, multi-temperature X-ray diffraction studies were conducted, starting at 100 K. On slow heating the occupation of the minor *endo*-nitrito component indeed started to increase, as shown in Fig. 4 ▸, reaching *ca* 5% at 160 K. At 180 K in addition to the *endo*-nitrito form some fraction of the *exo*-nitrito linkage isomer became spontaneously apparent, whereas the total amount of metastable-state species remained at the 5% level. In turn, upon further heating both metastable-state populations diminished, and completely vanished at 240 K (at this temperature only the nitro ground-state isomer was observed). The results confirm that the nitro-to-nitrito conversion is, to some extent, stimulated thermally, though the process is reversible (one crystal survived many cycles of experiments – including the trial ones – with no noticeable fatigue).

### 3.2. Solid-state spectroscopy   

Prior to photocrystallographic studies a set of solid-state infrared (IR) spectroscopic experiments were conducted. These were carried out to determine the optimal conditions of the examined photoisomerization reaction in the solid state and to estimate achievable conversion in the case of a thin-film sample. Firstly, the temperature scans were performed. Based on the IR spectra collected every 10 K, starting at room temperature down to 10 K, it was evident that no phase transition took place, though some subtle variations in band intensities were noted. Subsequently, a systematic scan of available LED excitation wavelengths in the 365–700 nm range was carried out at various temperatures. Each time IR absorption spectra were measured before and after sample irradiation.

**Figure 5:**
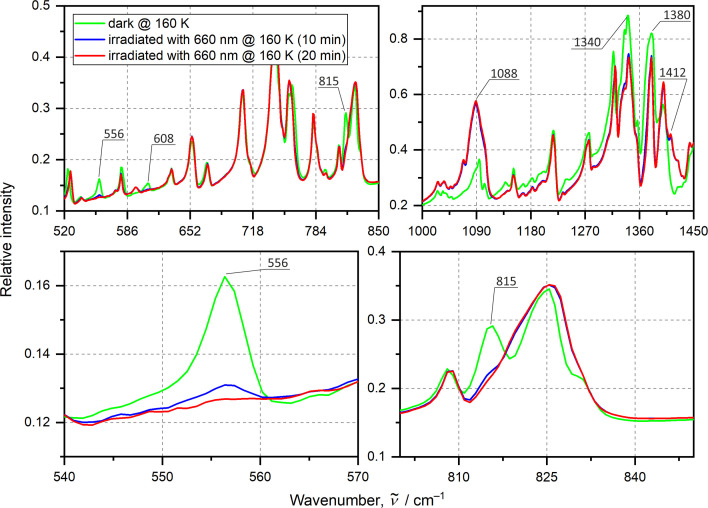
IR absorption spectra for the QTNiNO_2_ complex in the solid state recorded at 160 K. As indicted in the legend the green solid line denotes the ground-state structure, and the blue and red lines represent spectra recorded after 10 and 20 min after LED (660 nm centre wavelength) irradiation, respectively. Bottom panels show the 556 and 815 cm^−1^ bands [wagging ω(NO_2_) and scissoring δ(NO_2_) modes, respectively] behaviour in more detail.

The most significant changes were observed in the spectral ranges 500–850 cm^−1^ and 1000–1450 cm^−1^, involving mainly the NO_2_-related vibrational bands. The most striking observation was the complete disappearance of three vibrational bands at 556, 608 and 815 cm^−1^ after photoexcitation with a 530–660 nm LED light for about 10–20 min (Fig. 5 ▸). These observations are temperature dependent, *e.g.* at 100 K a significant decrease is observed, but the bands do not vanish completely within the 20 min of irradiation time. On the other hand, at 160 K the three bands have been completely erased within this time span. This is a clear hint that the achievable population of the light-induced species is temperature dependent [Fig. S2]. These findings also indicate that 160 K should be the optimal temperature for further photocrystallographic experiments. Through the analysis of similar literature examples (Chattopadhyay *et al.*, 2005 ▸; Chao *et al.*, 2012 ▸; Das *et al.*, 1998 ▸; Hortalá *et al.*, 2003 ▸; Laskar *et al.*, 2000 ▸; Ribas *et al.*, 1984 ▸; Hitchman & Rowbottom, 1982 ▸) supported by the isolated-molecule computations of harmonic normal-mode frequencies conducted for the molecule (see the supporting information), it was possible to identify and assign two of these bands (note the computed values tend to be overestimated). It appears that the 556 cm^−1^ band can be assigned to a wagging mode, ω(NO_2_), of the nitro ground-state form (570.7 cm^−1^ from computations), which is not present when the NO_2_ moiety binds to the metal centre via the oxygen atom, as supported by the simulation. Thus, the disappearance of this band clearly indicated nearly full conversion achieved during the LED irradiation. The band at 815 cm^−1^ corresponds to a scissoring vibrational mode, δ(NO_2_), of the nitric moiety. Our computations suggested this band should undergo a shift by about 8.5 cm^−1^ to higher frequencies (*i.e.* from 839.0 to 848.5 cm^−1^) when the NO_2_ group is transformed from its N-bound nitro to the O-bound nitrito form. A similar observation for this band was made by Warren *et al.* via Raman spectroscopy for another nickel nitro complex (Warren *et al.*, 2009 ▸). Indeed, after sample irradiation the δ(ONO) deformation mode has probably shifted and merged with other bands that are not influenced by the light irradiation of the sample. Furthermore, we observed a decrease of the bands corresponding to the symmetric ν_s_ (NO_2_) and asymmetric ν_as_ (NO_2_) stretching vibrations of the NO_2_ ligand at 1340 and 1380 cm^−1^ (1393.5 and 1502.9 cm^−1^ in theory, respectively). New bands appeared at 1088 and 1412 cm^−1^ which, according to the literature (and supported by our computations; 1079.4 and 1517.1 cm^−1^, respectively), most likely correspond to the ν(N—O) and ν(N=O) vibrations of the nitrito configuration, respectively. However, it should be noted that, based on the IR spectra collected and owing to the strong overlap of vibrational bands in the range 1200–1500 cm^−1^, it was not feasible to distinguish between various types of nitrito metastable states which could have been formed.

Irradiation of the sample with the 660 nm centre-wavelength LED seems to be less effective compared with the 530 or 590 nm LEDs. Despite its higher power, more time was needed to achieve full conversion judging by the disappearance of the 556 cm^−1^ band (compare Figs. 5 ▸ and S2). However, this wavelength was further used in the photocrystallographic experiments. Such choice was dictated by the fact that 660 nm is located at the tail of the UV–vis absorption spectrum collected for this sample (Fig. S3), which enhances light penetration of the crystal and prevents its rapid destruction (Hatcher *et al.*, 2019 ▸). We note here that the majority of IR experiments were performed on the same sample (see Fig. S2). No noticeable fatigue was observed, while all important spectroscopic features were reproducible confirming the transformation reversibility (the switch between nitro and nitrito states can be repeated multiple times).

### 3.3. *In situ* photocrystallography   

**Figure 6:**
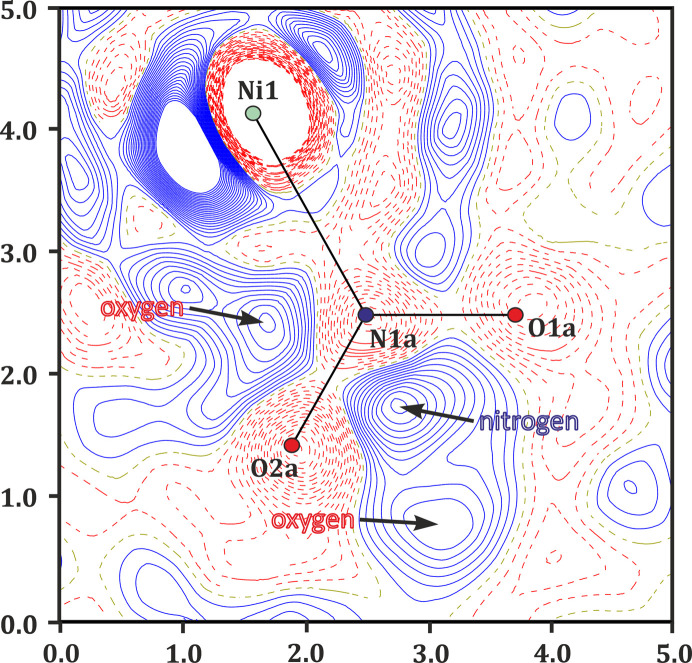
Photodifference map showing light-induced structural changes at 160 K for the QTNiNO_2_ crystal (for clarity only the nitro form of the light-OFF structure is shown; large arrows indicate peaks related to approximate positions of the metastable-state form atoms: upper arrow – oxygen atom of the *endo*-nitrito form, middle – nitro­gen atom, bottom – terminal oxygen atom of the *exo*-nitrito form; blue solid lines – positive values, red dashed lines – negative, contours at ±0.13 e·Å^−3^).

Photoswitchable properties of the studied system were finally examined photocrystallographically using our home-made light-delivery device allowing *in situ* experiments on a laboratory diffractometer (Kamiński *et al.*, 2016 ▸). Such experiments were especially important here, since based on the IR spectra it is problematic to distinguish, for example, between the *endo*- and *exo*-nitrito metastable isomers which may be generated in the course of the photoreaction. Therefore, after the multi-temperature X-ray diffraction studies in the dark, the crystal was cooled again to 160 K, irradiated for a period of 3 h with the 660 nm LED and subjected to a temperature scan. The formation of both *endo*-nitrito and *exo*-nitrito states was evident from the photodifference map immediately after irradiation at 160 K (Fig. 6 ▸). The *endo*- and *exo*-nitrito forms were populated up to about 14 and 10%, respectively. Temperature increase to 180 K did not change this picture and the populations remained essentially constant within experimental error. Small decreases of the metastable-state populations were observed at 200 K, at which they amounted to 13 and 9%, correspondingly. In turn, at 220 K a dramatic change was noted: only the *endo*-nitrito form prevailed (*ca* 3%) with no trace of the *exo*-nitrito isomer. It appears that in the 160–220 K temperature range the difference between the estimated populations of both metastable forms remains constant (around 4%). This suggests that both nitrito forms relax at approximately the same temperature with a similar rate. It is also worth noting that the ground-state and excited-state populations at 220 K are very comparable to those observed during the multi-temperature scan in the absence of light, which indicates that the irradiation effects prevail only up to about 200 K. Additionally, the (photo)crystallographic results are consistent with the spectroscopic findings, which show traces of the nitrito form up to around 230 K (Fig. S4).

It is also worth looking at the volume changes of the reaction cavity along with temperature in the dark (*i.e.* not irradiated) crystal structure and after irradiation. (Tables S5 and S6). During multi-temperature experiments performed in the absence of light one can see that the reaction cavity volume generally increases with temperature by *ca* 1.5 Å^3^, from 34.2 Å^3^ at 100 K to 35.7 Å^3^ at RT; the rise is approximately linear. Hence, it can be concluded that the dark structure reaction cavity volume increases with temperature as a consequence of simple thermal expansion of the entire crystal. At 160 K the cavity volume amounted to 34.9 Å^3^, yet after irradiation at this temperature the cavity volume increased by about 0.6 Å^3^. Further temperature rise resulted again in a slight increase of the cavity volume up to 220 K, at which a drop occurred, and then at 240 K the cavity volume appeared to be essentially the same size as it was at this temperature in the case of the non-irradiated crystal. Therefore, at 160 K the light-excitation will most likely produce larger populations of metastable states which leads to a cavity volume increase above its regular value at this temperature, whereas further elevation of temperature results in the structure relaxation towards its ground state (depopulation of metastable states). However, note the above conclusions must be treated rather qualitatively. The estimated reaction cavity volume standard deviation amounted to about 0.5 Å^3^ (Kutniewska *et al.*, 2019 ▸), and the reported changes are within this range.

### 3.4. Computational analysis   

**Figure 7:**
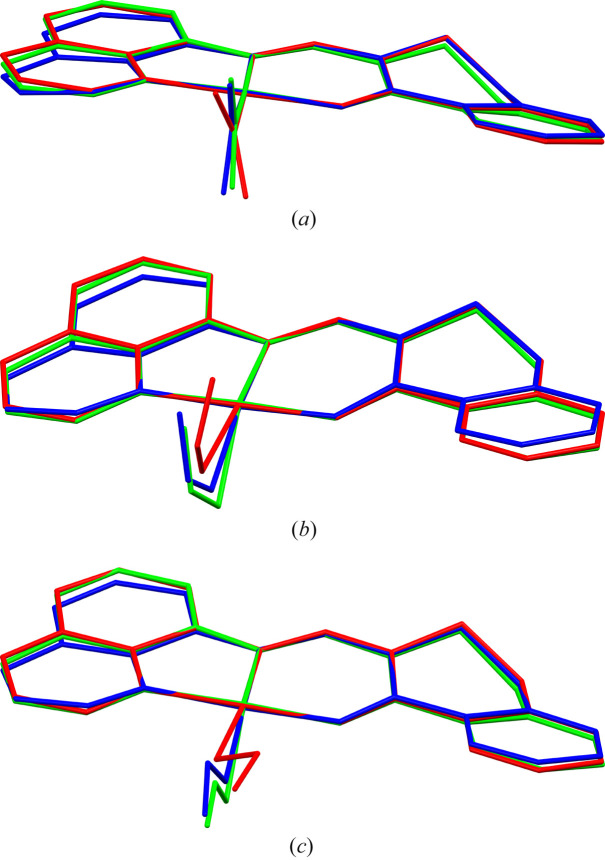
Overlay of complex geometries obtained from experiment (180 K dark dataset) (green) and theory – isolated molecule (red) and QM/MM (blue) optimizations. Geometries are compared for three states: (*a*) nitro ground state, (*b*) *endo*-nitrito and (*c*) *exo*-nitrito metastable states (molecules are rotated differently to emphasize the difference in NO_2_ ligand configurations; hydrogen atoms have been omitted for clarity; note the Ni1, O3, N2 and N3 atoms are superimposed via least-squares procedure).

In theoretical investigations that support the photocrystallographic analyses, the crystal environment has to be taken into account. For such purpose, geometry optimizations of various observed NO_2_-ligand binding modes, for both isolated molecules and molecules encapsulated in a crystal, were conducted. In the latter approach the central molecule is modelled with QM, whereas the crystal-field effects are modelled with MM. It has already been proven that the so-called QM/MM method is capable of describing the crystal environment reasonably well, and comprises a reliable theoretical support for photocrystallographic findings (Kutniewska *et al.*, 2019 ▸; Jarzembska *et al.*, 2014 ▸; Makal *et al.*, 2011 ▸; Benedict *et al.*, 2011 ▸). The isolated-molecule and QM/MM optimization results are compared in Fig. 7 ▸. The QM/MM geometries closely resemble the experimentally derived ones, much more accurately than the optimized isolated molecules do. Although the ligand geometry is rather well reproduced in all cases, the orientation of the NO_2_ fragment differs depending on whether the crystal environment is taken into account or not. Firstly, the nitro group in the ground-state molecule is notably more tilted in the optimized isolated molecule (70.31°; angle between two least-squares-fitted planes: Ni1, O3, N2, N3 and N1, O1, O2) than in the experimental (84.4°) and QM/MM (88.28°) results. Similar deviations are observed for the optimized metastable-state species. For the *endo*-nitrito isomer the N2−Ni1−O2 angle is equal to 175.56° in the isolated-molecule geometry, 165.80° in the QM/MM-derived molecule and 149.7(11)° in the experimental structure. This shows that the *endo*-nitrito NO_2_ group in the case of the isolated molecule tends to be more out of the plane of the organic ligand than is observed for the experimental or QM/MM geometries. Also, the QM/MM and experimental geometries of the *exo*-nitrito form match well. The angle between the organic and NO_2_ ligands least-squares planes (defined above) is equal to 74.85° and 86.5°, respectively. For the isolated-molecule geometry the ONO ligand is rotated significantly (37.18°) as a result of an extra intramolecular interaction formation, C1−H1⋯O2, which is absent in the crystalline state (instead numerous intermolecular contacts are formed). Together these confirm that the crystal environment has a profound influence on the geometry of all the isomeric forms of the studied complex and that, purely geometrically speaking, the NO_2_ ligand is quite labile in each isomeric form. The final molecular geometry constitutes a compromise between its thermodynamically optimal geometry and the strength of intermolecular interaction in the crystal structure.

Having obtained different molecular geometries, one may analyse the relative energetic stability of various linkage isomers of the QTNiNO_2_ complex. Relative energy values for all three computed linkage isomers are presented in Table 3 ▸. When comparing the isolated molecule optimization and QM/MM results the energetic trends are essentially the same, *i.e.* the nitro form is the most stable and the *exo*-nitrito isomer is the least stable. Nevertheless, there are significant differences regarding the energy values. In the case of the QM/MM results, the energetic differences between the isomers are more emphasized (+18.0 and +31.2 kJ mol^−1^, for the *endo*- and *exo*-nitrito forms, respectively) than for the isolated molecule. A similar conclusion was drawn in our previous study (Kutniewska *et al.*, 2019 ▸), but there the crystal confining effect was less pronounced. In addition, the above analysis confirms the validity of our refined structural model of all existing ground and metastable states.

Finally, a deeper insight into light-induced NO_2_ ligand transformations can be accomplished by the analysis of differences in intermolecular interactions stabilizing structural motifs when the molecule switches between various states. For that reason dimer interaction energies including both *endo*- and *exo*-nitrito linkage isomers were computed. Numerical results are presented in Table S7. For the purpose of computations the 180 K crystal structure determined after light irradiation was used, since it contains all the forms under consideration with relatively high populations, thus the refined disorder model was assumingly most reliable in that case. Computed interaction energies for the ground-state dimers are very well comparable with those presented previously for the 100 K geometries. Only slight differences (up to *ca* 0.8 kJ mol^−1^) are observed, which we ascribe to the differences in geometry resulting from the thermal and light-induced expansion of the crystal. Generally, for all **N**-type dimers, in which only one ‘internal’ (*i.e.* closer to the dimer centre; see Figs. 2 ▸ and 3 ▸) NO_2_ group was set switched to the metastable state, the presence of the *exo*-nitrito form results in decreased dimer stabilization compared with the analogous motif with the *endo*-nitrito isomer. The energy differences range from 1.6 to 6.4 kJ mol^−1^ in favour of the *endo*-nitrito state. In addition, for the **N2** dimer, where two ‘internal’ NO_2_ groups may undergo transformation, the switch of one group causes more destabilization to the motif than when it happens to the other. In this case the effect is larger when the group engaged in two hydrogen bonds is affected (3.2 versus 1.6 kJ mol^−1^). Interestingly, the **S1** dimer breaks the pattern. In this case the *exo*-nitrito dimer appears to be more energetically favoured than the *endo*-nitrito one (by about 2.6 kJ mol^−1^). When compared with the ground-state dimer, where the energy stabilization caused by the formation of new interactions (as described previously) is noted, the difference between the *endo*- and *exo*-nitrito forms is more subtle (only one terminal oxygen changes its position). Therefore, in this case we ascribe the energy differences to be the result of the electronic structure differences of both linkage isomers.

The above energetic analysis shows that the *exo*-nitrito form is the least stable (Table 3 ▸), while its presence also leads to some weakening of the intermolecular interactions in the crystal structure when compared with the *endo*-nitrito and nitro linkage isomers. In turn, the nitro isomer is the most energetically favourable. Hence, the obtained energetic trends somewhat explain the experimental observations, *i.e.* the fact that the ground-state structure mainly contains the nitro form, while the *endo*-nitrito form is more readily formed than the *exo* analogue upon temperature increase and/or light-irradiation. The *exo*-nitrito species can be seen in the crystal structure at some temperature range probably due to, in part, the thermal expansion of the system providing a larger available reaction cavity volume. Nevertheless, given only the enthalpy term, we cannot derive the full thermodynamic picture characterizing the relations between all free forms. In addition, one cannot neglect the kinetics of the whole process, which at high temperatures may result in very short lifetimes of the excited-state species. To explore these issues further we plan to carry on extensive computations of the isomerization reaction pathway following the literature (Skelton *et al.*, 2015 ▸; Warren *et al.*, 2014 ▸).

## 4. Summary and conclusions   

A new air-stable complex, QTNiNO_2_, in which the nickel centre is coordinated by a (N,N,O)-chelating ligand and the nitro moiety, undergoing photoisomerization reaction in the solid state, is reported. The crystal structure of QTNiNO_2_ determined at 100 K confirmed that the NO_2_ moiety exists majorly in the nitro form (η^1^-N(O)_2_); however, a tiny fraction (*ca* 2%) of the *endo*-nitrito (η^1^-ONO) isomer is also present. The NO_2_ group is involved in numerous relatively weak hydrogen-bond-like interactions, while the whole structure is also stabilized by effective π⋯π stacking interactions involving the aromatic moieties. The formation of the nitrito linkage isomer can be stimulated either thermally, or more effectively, by light irradiation. The highest conversion level, reaching 100%, for this system in the solid state was achieved at 160 K after exposure of a thin-film sample to 530–660 nm light, as indicated by the disappearance of the 556 cm^−1^ band in the IR spectrum. This band was ascribed to the ω(NO_2_) wagging vibrational mode active only in the ground-state N-bound nitro moiety. Nevertheless, using the IR technique it was not possible to determine whether the *endo*- or *exo*-nitrito isomer was formed during the sample exposure to LED light. X-ray diffraction multitemperature and *in situ* photocrystallographic experiments showed that, within a narrow temperature range, some fraction of the *exo*-nitrito form was present (around 180–200 K in the absence of light, 160–200 K for the irradiated sample). The population of the *exo*-nitrito isomer was always significantly lower than that of the *endo* equivalent. This can be explained at least by a generally lower energetic stability of the *exo*-nitrito isomer and the weaker intermolecular interactions it forms in the crystal structure when compared with the nitro and *endo*-nitrito linkage isomers, as indicated by the DFT computational results. It should also be noted that the total population of the metastable-state species in the crystal structure did not exceed 25%, which is, among others, the result of the limited light-penetration of the crystal.

Both the IR spectroscopic and X-ray diffraction studies confirmed that the light-induced metastable-state form is stable up to around 220–230 K, which is a relatively high temperature with respect to other reported photoswitchable nickel nitro coordination compounds in the literature (Warren *et al.*, 2009 ▸, 2014 ▸, 2012 ▸; Hatcher *et al.*, 2011 ▸; Hatcher, Bigos *et al.*, 2014 ▸). Furthermore, the process is reversible. Taking into account also the 100% conversion exhibited by the studied system in the form of a thin film, its low price, and convenient and robust synthesis, the results appear to be very promising in the context of future design of applicable photoswitchable materials. Hence, we plan to apply various modifications to the (N,N,O) ligand in order to deeply and systematically explore the effects of electronic and steric factors on the NO_2_-switching process and the stability of the generated metastable-state.

## Supplementary Material

Crystal structure: contains datablock(s) 100K_dark, 120K_dark, 140K_dark, 160K_dark, 160K_irr_160K, 180K_dark, 180K_irr_160K, 200K_dark, 200K_irr_160K, 220K_irr_160K, 240K_dark, 240K_irr_160K. DOI: 10.1107/S205225252001307X/lq5032sup1.cif

Structure factors: contains datablock(s) 100K_dark. DOI: 10.1107/S205225252001307X/lq5032sup2.hkl

Structure factors: contains datablock(s) 120K_dark. DOI: 10.1107/S205225252001307X/lq5032sup3.hkl

Structure factors: contains datablock(s) 140K_dark. DOI: 10.1107/S205225252001307X/lq5032sup4.hkl

Structure factors: contains datablock(s) 160K_dark. DOI: 10.1107/S205225252001307X/lq5032sup5.hkl

Structure factors: contains datablock(s) 180K_dark. DOI: 10.1107/S205225252001307X/lq5032sup6.hkl

Structure factors: contains datablock(s) 200K_dark. DOI: 10.1107/S205225252001307X/lq5032sup7.hkl

Structure factors: contains datablock(s) 240K_dark. DOI: 10.1107/S205225252001307X/lq5032sup8.hkl

Structure factors: contains datablock(s) 160K_irr_160K. DOI: 10.1107/S205225252001307X/lq5032sup9.hkl

Structure factors: contains datablock(s) 180K_irr_160K. DOI: 10.1107/S205225252001307X/lq5032sup10.hkl

Structure factors: contains datablock(s) 200K_irr_160K. DOI: 10.1107/S205225252001307X/lq5032sup11.hkl

Structure factors: contains datablock(s) 220K_irr_160K. DOI: 10.1107/S205225252001307X/lq5032sup12.hkl

Structure factors: contains datablock(s) 240K_irr_160K. DOI: 10.1107/S205225252001307X/lq5032sup13.hkl

Animated GIF files for selected harmonic vibrational modes. DOI: 10.1107/S205225252001307X/lq5032sup14.zip

Supporting information file. DOI: 10.1107/S205225252001307X/lq5032sup15.pdf

CCDC references: 1975196, 1975197, 1975198, 1975199, 1975200, 1975201, 1975202, 1975732, 1975733, 1975734, 1975735, 1975736

## Figures and Tables

**Table 1:** Selected crystal structure parameters for the studied QTNiNO_2_ complex The representative 100 K data collection; for more details see the supporting information.

Moiety formula	C_20_H_15_NiN_3_O_3_
Moiety formula mass *M*_r_ (a.u.)	404.04
Crystal system	Tetragonal
Space group	*I*4_1_ *a* (No. 88)
*Z*	16
*F*_000_	3328
Crystal colour, shape	Brown, prism
*a* (Å)	14.7759 (3)
*b* (Å)	14.7759 (3)
*c* (Å)	29.8577 (7)
*V* (Å^3^)	6518.7 (2)
*R*[*F*] [*I* > 3σ(*I*)] (%)	3.82
*R*[*F*] (all data) (%)	4.12
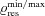 (e Å^−3^)	−0.48/+0.39

**Table 2:** Selected dimeric motifs present in the crystal structure of QTNiNO_2_ complex at 100 K: geometrical parameters and interaction energy values [*E*_int_; DFT(B3LYP)/6-311++G** level of theory] Geometries with the X−H neutron-normalized distances were used for computations.

Motif	*E*_int_ (kJ mol^−1^)	Selected interactions†	*d*_*D*–H_ (Å)	*d*_H⋯*A*_ (Å)	*d*_D⋯*A*_ or *d*_π⋯π_ (Å)	θ_*D*–H⋯*A*_ (°)
**S1**	−84.4	C9(π) ⋯C9(π)^#1^			3.645 (2)	
		C2(π) ⋯C8(π)^i^			3.562 (3)	
**N1**	−127.9	C12−H12a⋯O1a^ii^	0.96	2.76	3.164 (2)	106.17
		C12−H12b⋯O1a^ii^	0.96	2.87	3.164 (2)	98.95
		C20(π) ⋯C20(π)^ii^			3.549 (2)	
		C7(π) ⋯N3(π)^ii^			3.530 (2)	
		C8(π) ⋯C9(π)^ii^			3.556 (2)	
**N2**	−43.8	C17−H17⋯O1a^iii^	0.96	2.61	3.131 (2)	114.38
		C2−H2⋯O2a^iv^	0.96	2.50	3.126 (3)	122.54
		C3−H3⋯O2a^iv^	0.96	2.53	3.136 (2)	120.94
**N3**	−17.2	C15−H15⋯O1a^v^	0.96	2.65	3.495 (3)	146.75
		H12a⋯H15^v^			2.52‡	
**N4**	−19.9	H1⋯H13a^vi^			2.14‡	

† It should be noted that the given interaction energy values describe the total interaction between the two molecules comprising the structural motif, *i.e.* they include not only the hydrogen-bond(-like) contacts presented, but also some other weak interactions stabilizing the system.

‡ H⋯H contact. Symmetry operations: (i) − *x* + 1, − *y* + 1, − *z* + 1, (ii) − *x* + 1, − *y* + 1/2, *z*, (iii) *y* + 1/4, − *x* + 1.25, *z* + 1/4, (iv) − *y* +1.25, *x* − 1/4, *z* − 1/4, (v) *x* − 1/2, *y*, − *z* + 1.5, (vi) − *y* + 1.25, *x* + 1/4, − *z* + 1.25.

**Table 3:** Relative energy differences (Δ*E*) between ground (nitro) and metastable (*exo*- and *endo*-nitrito) states computed using the QM/MM approach and for the optimized isolated-molecule geometries Computations were performed at the DFT(B3LYP)/6-311++G** level of theory.

Form	Δ*E*_rel_ (kJ mol^−1^)	
	QM/MM	Isolated molecule
Nitro	0.0	0.0
*Endo*-nitrito	+18.0	+5.3
*Exo*-nitrito	+31.2	+11.9
