# Supplementary figures and images for: Photocrystallographic and spectroscopic studies of a model (N,N,O)-donor square-planar nickel(II) nitro complex: in search of high-conversion and stable photoswitchable materials
Source: IUCrJ. 2020 Oct 29;7(Pt 6):1188–98. doi: 10.1107/S205225252001307X (PMC7642791; doi:10.1107/S205225252001307X)

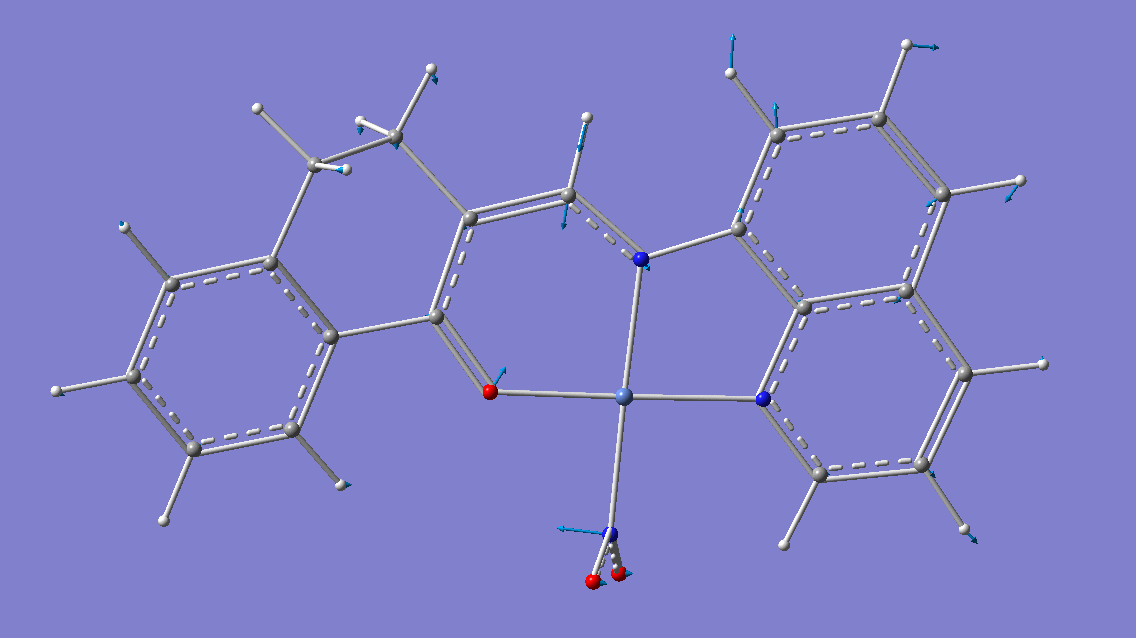

Supplement: Supplementary file 14 [file m-07-01188-sup14.zip › GS-nitro-mode33-570.71-wagging.gif]

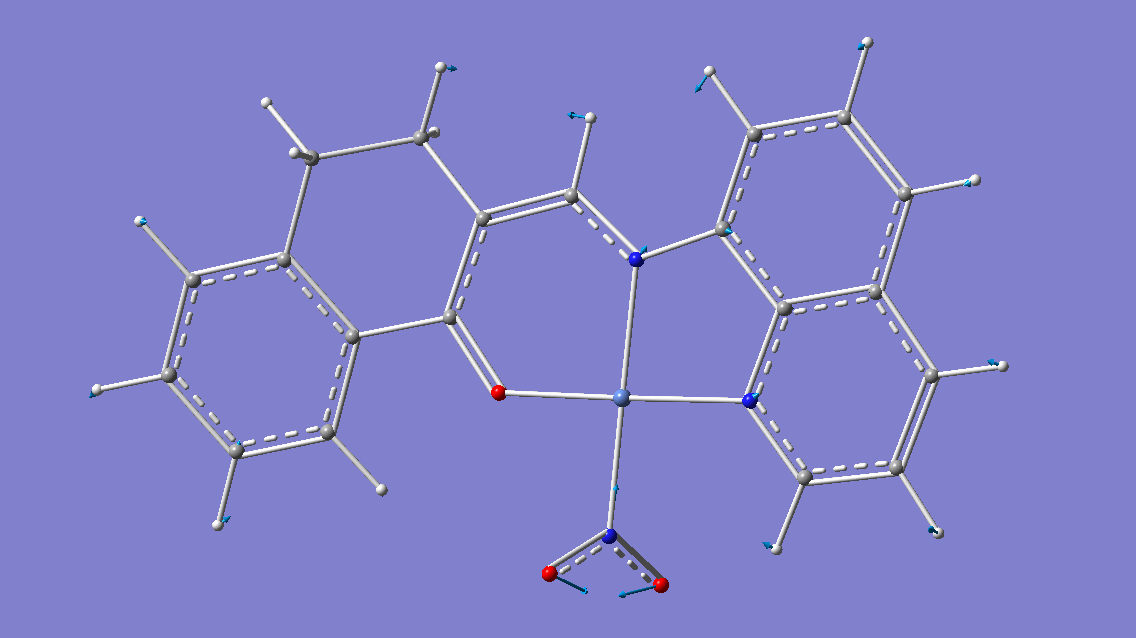

Supplement: Supplementary file 14 [file m-07-01188-sup14.zip › GS-nitro-mode51-839.02-scissoring.gif]

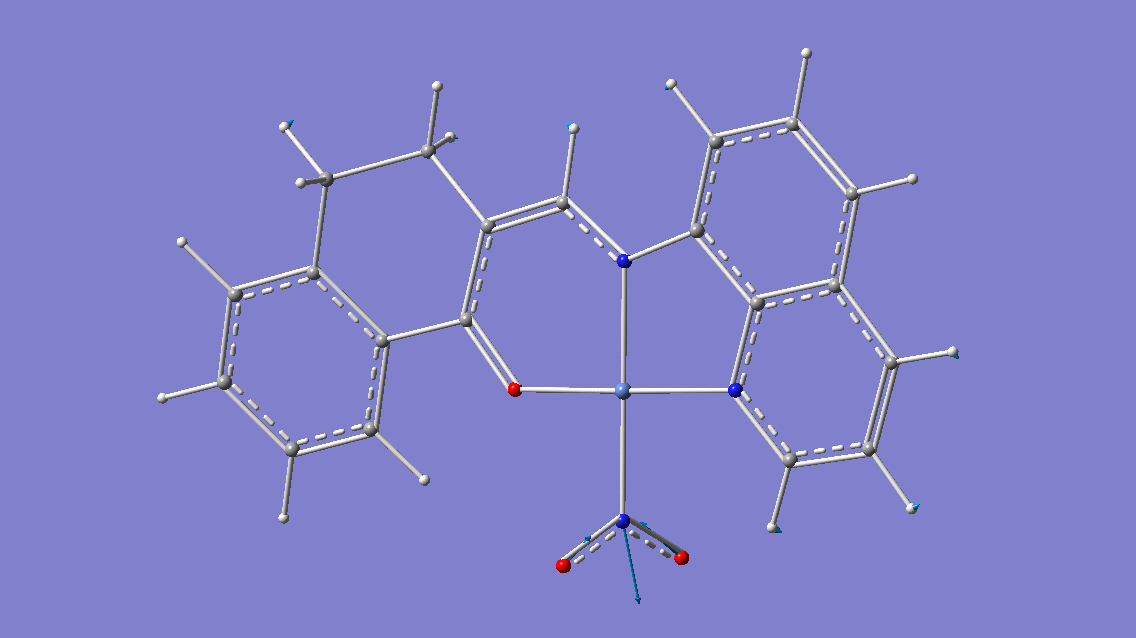

Supplement: Supplementary file 14 [file m-07-01188-sup14.zip › GS-nitro-mode87-1393.52-NO2sym.gif]

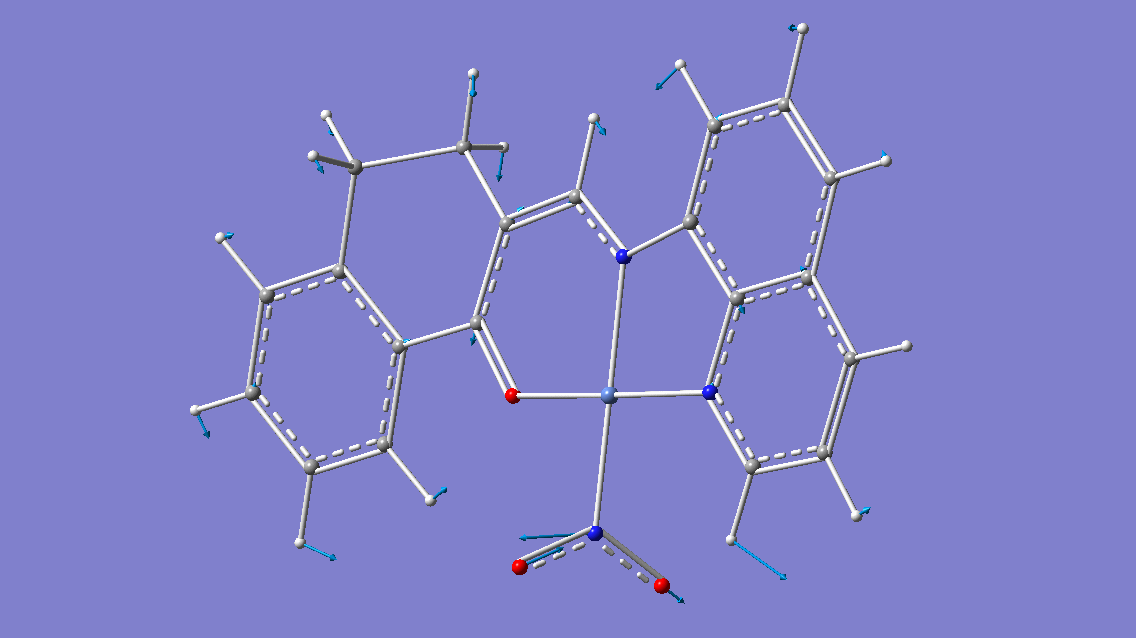

Supplement: Supplementary file 14 [file m-07-01188-sup14.zip › GS-nitro-mode96-1502.94-NO2asym.gif]

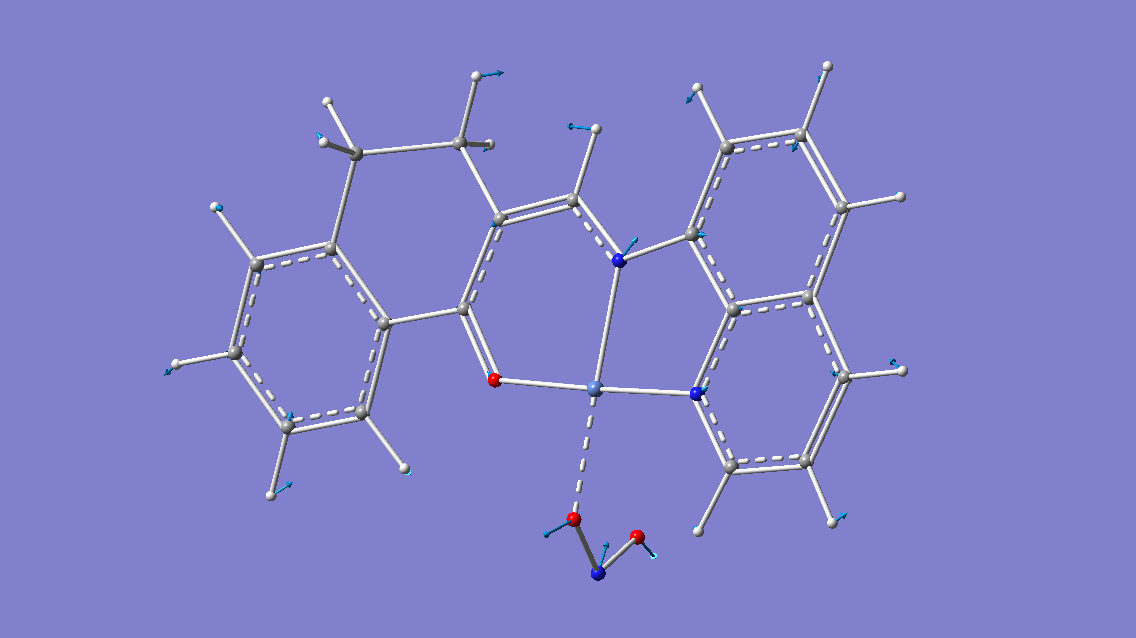

Supplement: Supplementary file 14 [file m-07-01188-sup14.zip › MS-endo-nitrito-mode51-847.50-scissoring.gif]

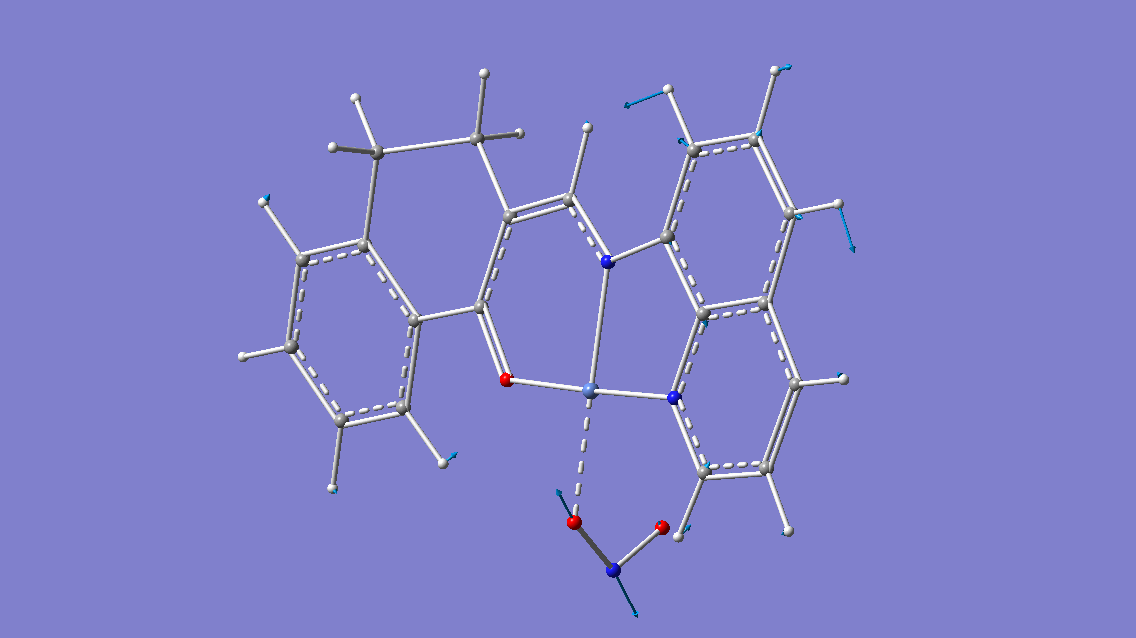

Supplement: Supplementary file 14 [file m-07-01188-sup14.zip › MS-endo-nitrito-mode67-1079.41-N-O.gif]

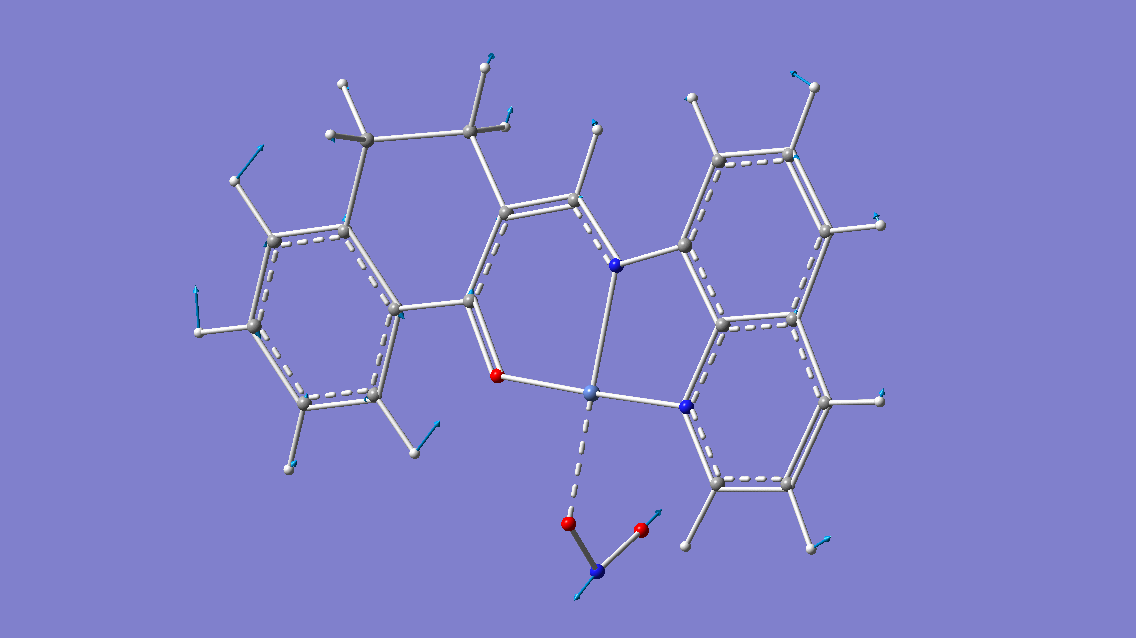

Supplement: Supplementary file 14 [file m-07-01188-sup14.zip › MS-endo-nitrito-mode98-1517.06-N=O.gif]
