# Supplementary material for: Photocrystallographic and spectroscopic studies of a model (N,N,O)-donor square-planar nickel(II) nitro complex: in search of high-conversion and stable photoswitchable materials
Source: IUCrJ. 2020 Oct 29;7(Pt 6):1188–98. doi: 10.1107/S205225252001307X (PMC7642791; doi:10.1107/S205225252001307X)
Supplement: Supplementary file 15 [file m-07-01188-sup15.pdf]

IUCrJ

Volume 8 (2021)

Supporting information for article:

Photocrystallographic and spectroscopic studies of a model (N,N,O)-donor square-planar nickel(II) nitro complex: in search of high-conversion and stable photoswitchable materials

Sylwia E. Kutniewska, Adam Krówczyński, Radoslaw Kamiński, Katarzyna N. Jarzembska, Sébastien Pillet, Emmanuel Wenger and Dominik Schaniel

S1. Extended experimental part

S1.1. Synthesis and crystal growth. The **QTNiNO2** compound was prepared using the literature procedure for the intermediate products (Dabrowski & Krówczyński, 1977), which were not isolated along the synthetic protocol (Scheme 1S). A mixture of 150 mg (1.0 mmol) of 1-tetralone in 150 mg (2.0 mmol) of ethyl formate was added to 30 mg (1.3 mmol) of molecular sodium in 20 mL of Et_2O, and stirred for 12 hours. After solvent evaporation the obtained hydroxymethyleneketone sodium salt was dissolved in 30 mL of MeOH, and then the 150 mg (1.0 mmol) of 8-aminoquinoline in 10 mL of MeOH was added, and then the mixture was neutralised with AcOH to pH ≈ 5. The semi-product, **QTH** (2-[8-quinolyloamino)methyl]-1-tetralone), was not isolated and its solution was brought to boil, followed by an addition of 300 mg (1.2 mmol) of $Ni(OAc)_2$ in 10 mL of MeOH. The solution was not heated anymore and continued to be stirred for the next 2 hours. The dark-brown mixture (containing **QTNiOAc** compound) was purified by filtration and the $LiNO_2$ salt in MeOH was added (*ca.* 0.5 mmol) (Whitaker *et al.*, 1995). The final product was cooled with an ice bath and filtered. Yield: 60% (0.6 mmol). ^1H NMR (500 MHz, $CDCl_3$) δ (ppm): 9.03 (s, 1H), 8.22 (d, 2H), 7.56 (m, 6H), 2.81 (d, 2H), 2.17 (s, 1H), 1.58 (s, 1H) (NMR spectrum was recorded at ambient temperature on a Varian NMR System 500 MHz spectrometer). Small brownish crystals of **QTNiNO2** suitable for single-crystal-X-ray diffraction were grown via diffusion-crystallisation method using *n*-hexane and MeOH solvents.

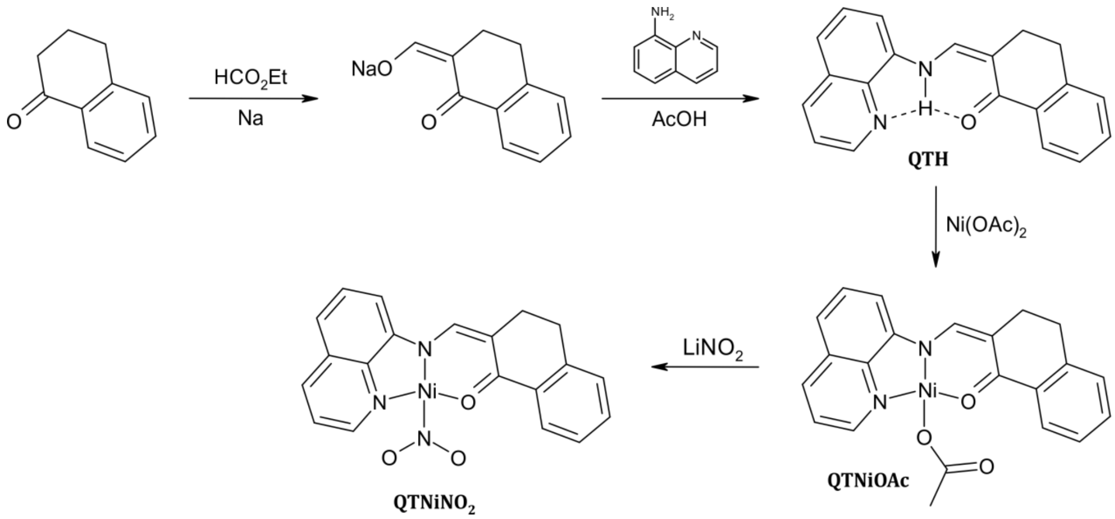

Scheme S1. The synthetic approach used to obtain the studied Ni^{II} complex **QTNiNO2**.

S1.2. X-ray diffraction. All X-ray diffraction data sets (including the trial ones) were collected using a Rigaku Oxford Diffraction SuperNova single-crystal diffractometer equipped with a CCD-type area detector, copper microfocus X-ray source (Cu-K$_\alpha$ radiation, λ = 1.54184 Å), 4-circle goniometer, multi-layer optics, and a low-temperature nitrogen gas-flow device by Oxford Cryosystems (700 Series Cryostream). Additionally, the diffractometer was equipped with the light-delivery device (Kamiński *et al.*, 2016), constructed by us and specifically re-designed to fit the SuperNova setup, allowing *in situ* photocrystallographic experiments. The device is based on collimating and focusing optics (fused-silica lenses), mounted inside the diffractometer enclosure, and fibre optics which guide light from the source located outside the enclosure. In the case described here, a fibre-coupled (Thorlabs UM22-400; multimode solarisation-resistant fibre of 400 µm core diameter) light-emitting diode (LED) of 660 nm central wavelength (Thorlabs M660F1) was used as the light source (LED was driven by our home-made controller). The optimal data-collection strategy took into account the light-delivery device mount and was prepared using the native diffractometer software (Rigaku Oxford Diffraction, 2020). The same strategy (in which only the exposure time was adjusted for various temperatures) was used for all data collections. All data collections were carried out in complete darkness (the sample mounting and centring was done at room temperature prior to any further data collections; all experiments were performed with all diffractometer lights permanently switched off). The overall experimental procedure for crystal was as follows: (i) crystal mounting at room temperature and cooling to 160 K at 360 K·s^{-1}, (ii) multi-temperature data collections at various temperatures ranging from 100 to 240 K with a step of 20 K (data sets' abbreviations: **100K-dark**, **120K-dark**, **140K-dark**, **160K-dark**, **180K-dark, 200K-dark, 240K-dark** (iii) cooling the crystal down to 160 K and light irradiation for about 180 minutes (during this time the crystal was continuously rotated to ensure uniform exposure; LED driving current was set to 800 mA), (iv) X-ray diffraction measurements at various temperatures ranging from 160 to 240 K with a step of 20 K (data sets' abbreviations: **160K-irr-160K**, **180K-irr-160K**, **200K-irr-160K**, **220K-irr-160K**, **240K-irr-160K**). Further data processing (*i.e.* unit-cell determination, raw diffraction frame integration, absorption correction, scaling) was common for all data collections. All structures were solved using an intrinsic phasing method, as implemented in the *SHELXT* program (Sheldrick, 2015), and refined with the *JANA* package (Petříček *et al.*, 2014) within the independent atom model (IAM)

approximation. The disordered structures were modelled using a standard splitting model, in which the initial positions of metastable-state atoms were determined from the residual or photodifference maps (Fournier & Coppens, 2014, Schmøkel *et al.*, 2010). Whenever possible, disordered parts were refined anisotropically, although for parts with lower occupancy it was not possible (in this cases atoms were treated as isotropic). For the *endo*-nitrito form the oxygen atom from the metastable state close to the one from the ground state is kept with identical coordinates as in the nitro form in all models. All the final refinement statistics are summarised in Table 1S. The CIF files are present in the Supporting Information, or can be retrieved from the Cambridge Structural Database (CSD) (Allen, 2002, Groom *et al.*, 2016) (deposition numbers: CCDC 1975196-202 & 1975732-736). Furthermore, sets of raw diffraction frames and associated data are available online under the following DOI: 10.18150/repod.5160503 (Repository for Open Data, Interdisciplinary Centre for Mathematical and Computational Modelling, University of Warsaw, Warsaw, Poland).

S1.3. Spectroscopy. All infrared (IR) absorption measurements were performed from 10 to 295 K using the Nicolet 5700 FT-IR spectrometer (spectral resolution of 2 cm^{-1} in the range of 360 – 4000 cm^{-1}) equipped with a closed-cycle cryostat (Oxford Optistat V01). The sample was grinded, mixed with spectroscopic grade KBr, pressed into pellets, and glued to the cold finger of the cryostat using silver-paste thermal adhesive. During measurements the sample was kept in vacuum inside the cryostat. Irradiation of the sample was achieved through the cryostat window using various LEDs (Thorlabs L and LP series), the central wavelength of which covered the range from violet to red (from 385 to 735 nm).

UV-Vis absorption spectroscopy measurements were performed with the CARY 4000 spectrometer in the 200 – 900 nm wavelength range. The sample, prepared in the same way as for the IR measurements, was mounted inside Oxford Optistat equipped with quartz windows, allowing to control the temperature from 10 to 300 K. The samples were irradiated through the cryostat windows using the same set of LEDs as used in the IR absorption experiments.

S1.4. Theoretical computations. All computations were carried out with the *GAUSSIAN* package (*GAUSSIAN16* version) (Frisch *et al.*, 2016). In the case of quantum-mechanics / molecular-mechanics (QM/MM) computations the model of a ground- or metastable-state molecule in a crystal environment (Kamiński *et al.*, 2010) composed of

a central molecule (QM part) and a shell (MM part) which was cut-out from the studied crystal structure with a radius of 12 Å (all C−H distances were set to the neutron-normalized values (Allen & Bruno, 2010, Allen *et al.*, 1987)). The semi-automatic generation of input files was accomplished with the *CLUSTERGEN* program (Kamiński *et al.*, 2013). The density functional level of theory was applied for the optimisation of the central molecule (DFT(B3LYP)/6-311++G** (Becke, 1988, Perdew, 1986, Lee *et al.*, 1988, Krishnan *et al.*, 1980, Clark *et al.*, 1983, McLean & Chandler, 1980)), whereas the molecular shell was kept fixed and approximated with the Universal Force Field (UFF) (Rappé *et al.*, 1992) employing Hirshfeld atomic charges (Hirshfeld, 1977) derived initially at the same level of theory, including both the functional and the basis set. Dimer interaction energies, the isolated-molecule geometry optimisations and normal-mode frequencies were also calculated at the DFT(B3LYP)/6-311++G** level of theory. For harmonic mode computations no imaginary frequencies were found. In the interaction energy computations the Grimme empirical dispersion correction (Grimme, 2006, 2004), modified by the Becke-Johnson damping function (Grimme *et al.*, 2010, Grimme *et al.*, 2011) and correction for BSSE (Boys & Bernardi, 1970, Simon *et al.*, 1996). were applied. The automatic generation of molecular motifs was accomplished with the *CLUSTERGEN* program (Kamiński *et al.*, 2013).

Table S1. Selected X-ray data collection, processing and refinement parameters for all presented crystal structures.#

Data set	**100K-dark**	**120K-dark**	**140K-dark**	**160K-dark**	**180K-dark**	**200K-dark**
Moiety formula	$C_{20}H_{15}NiN_3O_2$					
Moiety formula mass, M_r / a.u.	404.04					
Crystal system	tetragonal					
Space group	$I4_1/a$ (no. 88)					
Z	16					
F_{000}	3328					
Crystal colour & shape	brown prism					
Crystal size / mm^3	0.05×0.05×0.13					
T / K	100	115	140	160	180	200
a / Å	14.7759(3)	14.7908(3)	14.8076(3)	14.8239(4)	14.8394(3)	14.8584(3)
b / Å	14.7759(3)	14.7908(3)	14.8076(3)	14.8239(4)	14.8394(3)	14.8584(3)
c / Å	29.8577(7)	29.8489(7)	29.8449(8)	29.8395(8)	29.8320(8)	29.8280(8)
V / Å^3	6518.7(2)	6530.0(2)	6543.9(3)	6557.2(3)	6569.2(3)	6585.2(3)
d_{calc} / g·cm^{-3}	1.6468	1.644	1.6404	1.6371	1.6341	1.6302
θ range	3.34° – 74.90 °	3.33° –74.86°	3.33° – 74.79°	3.33° – 74.75°	3.33° – 74.87°	3.32° – 74.74°
Absorption coefficient, μ / mm^{-1}	1.963	1.9600	1.956	1.952	1.948	1.943
No. of reflections collected / unique	24214 / 3328	24459 / 3332	24452 / 3339	24646 / 3344	24635 / 3357	24686 / 3361
R_{int}	3.80%	3.87%	3.87%	3.85%	3.99%	3.89%
No. of reflections with $I > 3\sigma(I)$	3023	3040	3046	3030	2985	3015
No. of parameters / restraints / constraints	252 / 0 / 70	252 / 0 / 70	252 / 0 / 70	252 / 0 / 70	259 / 0 / 78	259 / 0 / 78

$R[F]$ $(I > 3\sigma(I))$	3.82%	3.87%	3.86%	3.85%	3.99%	3.89%
$R[F]$ (all data)	4.11%	4.17%	4.16%	4.20%	4.38%	4.24%
$\varrho_{res}^{min/max}$ / e·Å^{-3}	−0.48 / +0.39	−0.48 / +0.42	−0.45 / +0.36	−0.47 / +0.38	−0.47 / +0.37	−0.40 / +0.37
CCDC code	1975196	1975197	1975198	1975199	1975200	1975201

[#] All raw data are available under the following DOI: 10.18150/repod.5160503.

Table S1 (continued). Selected X-ray data collection, processing and refinement parameters for all presented crystal structures.

Data set	**240K-dark**	**160K-irr-160K**	**180K-1-irr-160K**	**200K-1-irr-160K**	**220K-1-irr-160K**	**24K-1-irr-160K**
Moiety formula						
Moiety formula mass, M_r / a.u.						
Crystal system						
Space group						
Z						
F_{000}						
Crystal colour & shape						
Crystal size / mm^3						
T / K	240	160	180	200	220	240
a / Å	14.8930(6)	14.8206(7)	14.8477(3)	14.8663(3)	14.8778(3)	14.8976(3)
b / Å	14.8930(6)	14.8206(7)	14.8477(3)	14.8663(3)	14.8778(3)	14.8976(3)
c / Å	29.8290(13)	29.8617(14)	29.8554(5)	29.8410(5)	29.8291(6)	29.8244(6)
V / Å^3	6616.1(5)	6559.1(5)	6581.7(2)	6595.1(2)	6602.6(2)	6619.2(2)
d_{calc} / g·cm^{-3}	1.6226	1.6367	1.631	1.6277	1.6259	1.6218
θ range	10.32° – 64.32°	10.36° – 64.39°	3.32° – 73.35°	3.32° – 73.26°	3.32° –74.61 °	3.32° – 74.62°
Absorption coefficient, μ / mm^{-1}	1.934	1.951	1.944	1.941	1.938	1.933
No. of reflections collected / unique	8960 / 2025	8900 / 2093	24148 / 3243	23884/ 3296	24641/ 3362	24754 / 3367
R_{int}	9.34%	4.99%	3.74%	4.15%	3.69%	3.75%
No. of reflections with $I > 3\sigma(I)$	1740	1772	2857	2946	3080	3050
No. of parameters / restraints / constraints	244 / 0 / 60	259 / 0 / 75	259 / 0 / 78	259 / 0 / 78	252/ 0 / 70	244 / 0 / 78

$R[F]$ $(I > 3\sigma(I))$	9.34%	4.99%	3.74%	4.15%	3.69%	3.78%
$R[F]$ (all data)	9.92%	5.80%	4.21%	4.23%	3.95%	4.02%
$\varrho_{\mathrm{res}}^{\mathrm{min/max}}$ / e·Å^{-3}	-0.86 / +0.90	−0.48 / +0.35	−0.58 / +0.41	−0.52 / +0.62	-0.33 / +0.40	−0.36 / +0.32
CCDC code	1975202	1975732	1975733	1975734	1975735	1975736

All raw data are available under the following DOI: 10.18150/repod.5160503.

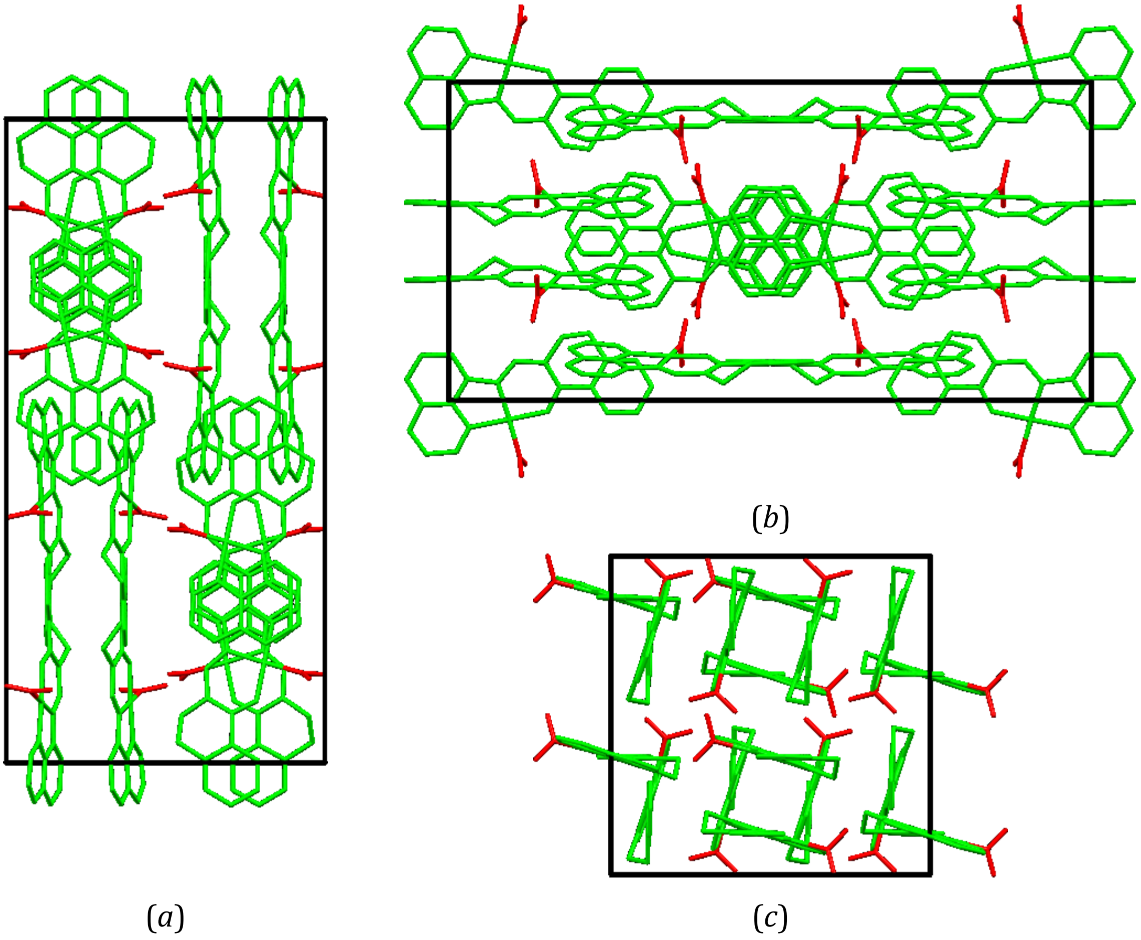

Figure S1. Crystal packing of the **QTNiNO$_2$** complex along the (*a*) [100], (*b*) [010], and (*c*) [001] directions. Organic ligands and nickel atoms are shown in green colour; NO_2 ligands are shown in red. Hydrogen atoms are omitted for clarity.

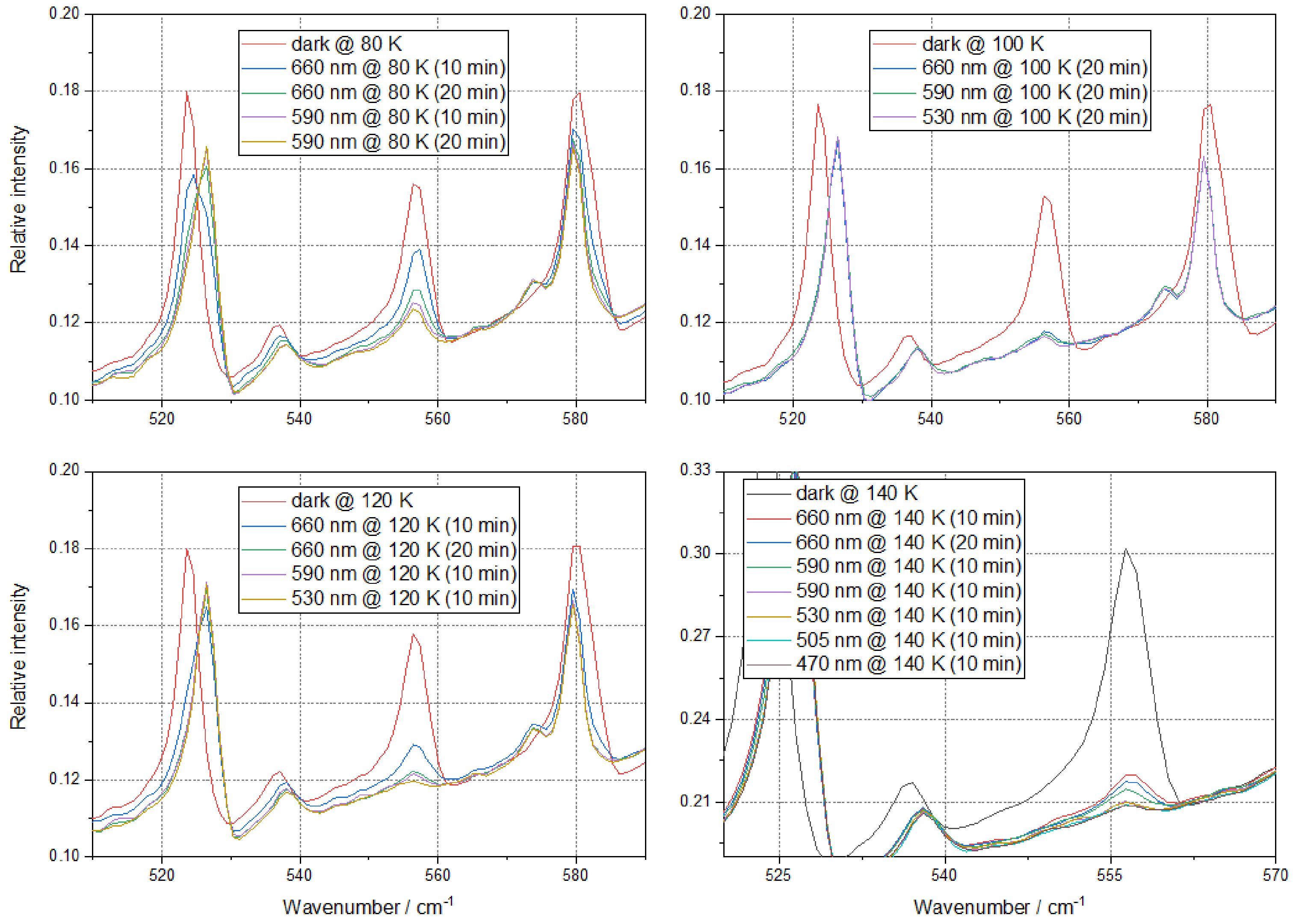

Figure S2. IR absorption spectra for the **QTNiNO$_2$** complex in the solid state recorded at various temperatures. For each plot legend indicates what kind of experiments was performed. Unless indicated otherwise the LED light source was used. Spectra concentrate on the 556 cm^{-1} band to show the temperature dependence of the effectivity of the irradiation.

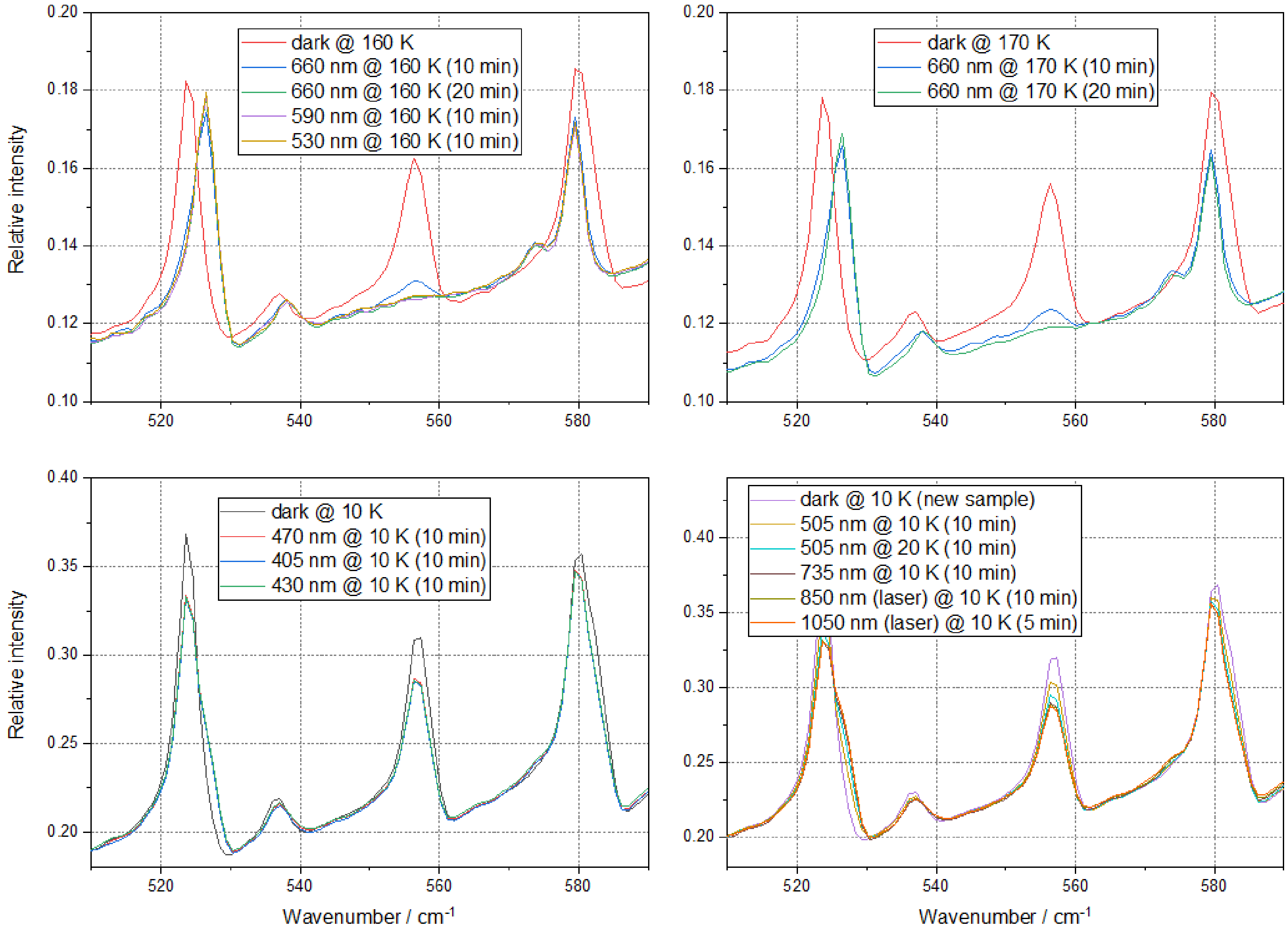

Figure S2 (continued). IR absorption spectra for the **QTNiNO$_2$** complex in the solid state recorded at various temperatures. For each plot legend indicates what kind of experiments was performed. Unless indicated otherwise the LED light source was used. Spectra concentrate on the 556 cm^{-1} band to show the temperature dependence of the effectivity of the irradiation.

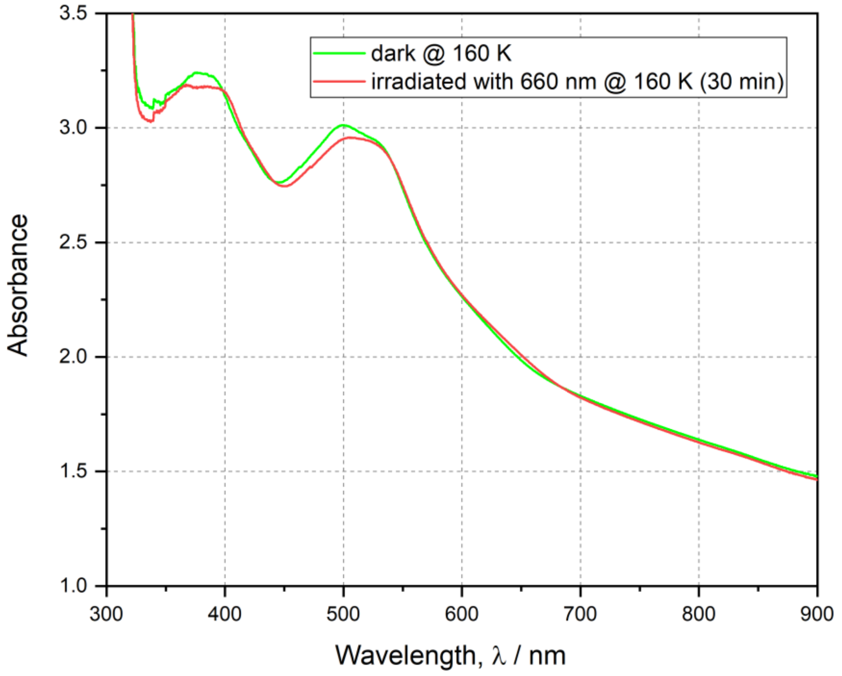

Figure S3. UV-Vis spectra collected for dark (green line) and irradiated (660 nm LED light, 30 min; red line) sample. Please note there are very little variations of the spectrum with temperature and irradiation and irradiation wavelength.

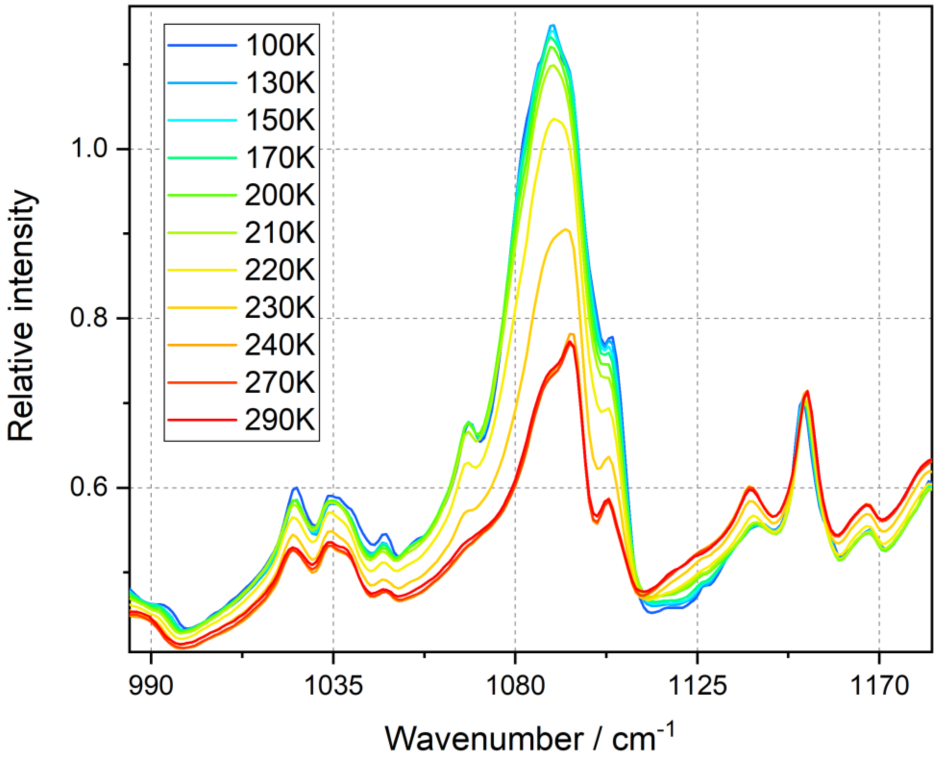

Figure S4. Multi-temperature IR spectrum recorded after irradiation (multi-LED 470-660 nm irradiation for total time of 110 min) showing intensity changes of the 1088 cm^{-1} peak. Intensity rise observed during the irradiation (see Figure 5) indicates the formation of the nitrito form. Heating experiments, when the intensity of this peak drops down, show the metastable nitrito form is present with some considerable population up to 220 K, begins to fade at around 230 K, while the sample returns to the ground state at 240 K.

Table S2. Motif interaction energies for dimers consisting of one or two molecules in the *endo*-nitrito metastable form. Computations based on the **100K-dark** data set geometries. For more information see Figure 3.

Motif	E_{int} / kJ·mol^{-1}			
	GS-GS	*GS-MS*	*MS-GS*	*MS-MS*
**S1**	−84.4	−86.9	−86.9	−88.9
**N1**	−127.9	−120.1	−120.1	−113.4
**N2**	−43.8	−48.4	−36.6	−41.1
**N3**	−17.2	−16.5	−9.6	−9.0
**N4**	−19.9	−17.3	−14.4	−11.2

Table S3. Experimentally-determined populations of the ground (nitro) and metastable (*endo*-nitrito and *exo*-nitrito) states at various temperatures for crystal during the temperature scan.

Data set	*Population, P%*		
	nitro<br>η^1-N(O)$_2$	*endo*-nitrito<br>η^1-ONO	*exo*-nitrito<br>η^1-(O)$_2$N
**100K-dark**	98.0(5)	2.0(5)	–
**120K-dark**	96.4(7)	3.6(7)	–
**140K-dark**	95.9(6)	4.1(6)	–
**160K-dark**	95.2(7)	4.8(7)	–
**180K-dark**	95.2(7)	3.6(8)	1.2(10)
**200K-dark**	96.0(6)	2.4(8)	1.5(10)
**240K-dark**	100	–	–

Table S4. Experimentally-determined populations of the ground (nitro) and metastable (*endo*-nitrito and *exo*-nitrito) states at various temperatures for crystal after irradiation at 160 K.

Data set	*Population, P%*		
	nitro<br>η^1-N(O)$_2$	*endo*-nitrito<br>η^1-ONO	*exo*-nitrito<br>η^1-(O)$_2$N
**160K-irr-160K**	75(1)	14(2)	10(2)
**180K-irr-160K**	74.6(8)	15(1)	11(1)
**200K-irr-160K**	77.7(9)	13(1)	9(1)
**220K-irr-160K**	97.2(6)	2.8(6)	–
**240K-irr-160K**	100	–	–

Table S5. Reaction cavity volumes calculated per one complex molecule (*i.e.* with the removed NO_2 group) calculated in the *MERCURY* program (probe radius of 1.2 Å, grid spacing of 0.1 Å) for non-irradiated crystals.

Data set	*Cavity volume per molecule*, V_{cav} / Å^3
**100K-dark**	34.21
**120K-dark**	34.36
**140K-dark**	34.60
**160K-dark**	34.88
**180K-dark**	35.03
**200K-dark**	35.25
**240K-dark**	35.68

Table 6S. Reaction cavity volumes calculated per one complex molecule (*i.e.* with the removed NO_2 group) calculated in the *MERCURY* program (probe radius of 1.2 Å, grid spacing of 0.1 Å) for irradiated crystals.

Data set	*Cavity volume per molecule*, V_{cav} / Å^3
**160K-irr-160K**	35.52
**180K-irr-160K**	35.78
**200K-irr-160K**	35.82
**220K-irr-160K**	35.30
**240K-irr-160K**	35.56

Table S7. Motif interaction energies for dimers consisting of one molecule in the *endo*- or *exo*-nitrito metastable form. Computations based on the **180K-irr-160K** data set geometries. For more information see also Figure 3 caption.

Motif	E_{int} / kJ·mol^{-1}				
	GS-GS	*GS-ENDO*	*GS-EXO*	*ENDO-GS*	*EXO-GS*
**S1**	−83.6	−83.5	−86.1	–	–
**N1**	−127.2	−120.4	−115.0	–	–
**N2**	−43.8	−45.7	−44.1	−38.7	−35.4
**N3**	−17.2	–	–	−12.4	−9.7
**N4**	−20.0	–	–	−16.2	−9.8

Table S8. Numerical values for wavenumbers and relative intensities of isolated-molecule-computed harmonic vibrations for three forms (nitro, *endo*-nitrito and *exo*-nitrito) for the studied **QTNiNO2** complex.

	Nitro form		*Endo-nitrito form*		*Exo-nitrito form*	
Mode No.	$\tilde{\nu}$ / cm^{-1}	ε / $M^{-1}\cdot cm^{-1}$	$\tilde{\nu}$ / cm^{-1}	ε / $M^{-1}\cdot cm^{-1}$	$\tilde{\nu}$ / cm^{-1}	ε / $M^{-1}\cdot cm^{-1}$
1	22.685	139.984	23.213	251.144	25.410	155.415
2	30.714	315.703	26.163	142.130	32.290	202.509
3	39.905	7.188	35.428	162.222	41.155	7.832
4	47.368	16.078	48.936	74.479	62.497	123.836
5	83.025	59.025	90.010	12.299	88.126	23.631
6	92.342	0.199	94.758	14.870	99.493	21.745
7	116.931	26.967	111.938	30.097	103.529	35.860
8	136.181	87.237	145.300	20.035	124.805	18.367
9	153.512	20.335	157.489	29.090	158.312	15.573
10	170.648	25.274	161.758	41.108	168.772	47.387
11	176.046	29.283	174.826	21.738	173.241	25.527
12	191.762	103.447	184.560	10.267	188.215	67.829
13	219.652	41.321	210.618	55.417	215.213	19.642
14	243.628	5.654	224.716	59.835	228.987	55.944
15	247.310	47.974	243.408	10.614	236.382	77.593
16	250.041	68.929	259.502	17.250	246.837	4.661
17	277.306	29.229	280.426	11.665	265.117	36.692
18	287.383	4.359	290.727	11.985	285.590	13.853
19	314.969	4.462	332.236	31.295	297.329	0.884
20	353.649	20.406	352.212	23.189	340.441	34.882
21	378.576	127.074	361.146	23.275	356.694	1.013
22	390.953	43.058	383.376	20.075	382.592	24.040
23	414.472	80.308	397.058	63.891	408.599	37.455
24	432.935	47.766	415.164	158.155	427.862	65.268
25	460.695	45.810	438.948	91.509	441.026	98.237
26	465.496	8.345	463.095	56.054	465.281	19.489
27	470.840	19.712	466.715	14.728	468.541	31.112
28	479.046	15.750	470.847	11.786	471.838	7.876
29	490.802	41.037	478.538	16.822	480.964	16.806
30	508.347	22.861	490.329	43.666	493.441	41.259
31	526.240	104.017	507.231	16.239	509.190	18.723

32	548.020	19.972	540.174	14.709	526.504	104.600
33	570.708	21.255	554.612	18.735	549.801	9.697
34	588.549	21.140	584.792	0.677	584.300	6.496
35	594.926	15.481	594.356	19.420	594.940	18.602
36	619.313	32.434	603.393	18.874	601.986	6.889
37	639.441	3.471	625.639	27.528	638.631	17.706
38	645.007	10.055	646.581	9.805	647.358	14.296
39	661.012	133.320	658.583	101.439	661.215	138.253
40	682.247	45.707	683.866	64.273	684.317	32.074
41	726.025	96.764	722.789	78.564	727.324	80.668
42	728.814	46.055	730.773	63.718	729.802	59.680
43	754.251	224.366	755.113	212.418	757.569	130.037
44	758.679	220.607	758.332	221.573	758.904	334.370
45	793.354	138.183	794.918	120.947	796.344	119.864
46	799.273	2.633	797.340	3.344	800.412	0.240
47	809.816	14.390	809.986	17.115	809.982	15.201
48	822.588	91.644	823.213	100.254	822.651	81.822
49	830.715	74.640	833.820	33.944	832.971	22.367
50	834.000	162.887	834.639	174.116	836.913	191.055
51	839.018	72.468	847.496	47.249	855.803	326.844
52	886.521	0.599	885.799	0.202	886.702	1.032
53	891.018	0.289	891.765	0.492	894.911	0.409
54	921.251	19.145	918.945	20.380	921.503	21.150
55	935.845	40.480	933.439	31.544	936.620	33.713
56	948.243	34.100	945.467	25.063	945.731	22.762
57	970.225	8.723	978.055	4.422	978.156	1.166
58	977.364	8.955	978.112	2.129	981.863	7.153
59	978.938	1.541	982.717	3.352	990.354	1.426
60	993.783	57.579	992.360	63.620	992.380	192.681
61	1002.817	0.664	1005.592	0.942	997.996	1921.908
62	1004.587	1.276	1005.928	0.485	1011.784	4.490
63	1042.843	134.918	1041.663	148.238	1014.134	2.670
64	1054.405	48.328	1054.235	42.848	1043.252	170.878
65	1067.001	5.626	1063.038	150.965	1053.716	47.284
66	1073.225	4.818	1066.740	26.092	1063.925	11.068
67	1099.331	1.430	1079.413	596.351	1067.933	4.746
68	1117.124	135.891	1095.342	59.991	1092.866	4.544
69	1129.191	68.295	1112.899	127.658	1115.722	132.000
70	1165.232	23.086	1128.658	74.062	1129.023	62.359

71	1184.897	62.502	1164.181	23.077	1165.150	21.658
72	1187.380	4.457	1184.794	60.471	1185.623	58.726
73	1201.522	27.930	1186.712	3.208	1187.393	2.283
74	1216.941	86.207	1200.651	26.230	1201.027	22.635
75	1220.627	5.957	1216.063	88.000	1216.984	98.917
76	1241.324	175.345	1220.790	5.010	1221.167	4.324
77	1246.691	19.906	1240.757	168.213	1242.072	160.089
78	1271.465	46.385	1245.479	19.198	1248.203	22.296
79	1287.874	184.538	1261.521	28.740	1261.334	40.409
80	1302.080	190.802	1286.546	201.953	1289.006	157.687
81	1314.627	322.503	1298.604	339.433	1301.612	301.876
82	1342.338	94.761	1313.630	349.592	1316.199	355.568
83	1354.317	455.605	1342.125	120.157	1342.329	115.262
84	1361.304	169.705	1353.962	760.406	1355.359	797.843
85	1367.317	1962.739	1360.272	237.360	1360.941	624.815
86	1382.743	78.016	1363.256	1557.757	1365.673	1019.795
87	1393.520	595.494	1381.886	61.079	1382.218	69.550
88	1405.783	549.396	1403.602	877.666	1403.150	822.827
89	1430.746	678.429	1425.821	184.723	1428.385	298.766
90	1457.771	528.157	1456.633	530.871	1459.427	97.152
91	1459.911	17.760	1459.681	96.616	1460.411	549.179
92	1473.824	112.192	1470.876	330.318	1472.286	240.031
93	1480.865	120.698	1479.701	196.128	1480.359	167.565
94	1489.035	266.097	1488.863	254.107	1488.961	209.828
95	1492.110	373.944	1491.722	606.196	1492.517	793.485
96	1502.941	1538.406	1513.536	206.633	1515.373	299.244
97	1516.290	75.067	1516.183	97.790	1517.377	121.081
98	1518.067	289.891	1517.058	772.126	1546.920	57.613
99	1549.020	69.106	1544.787	67.099	1584.315	787.310
100	1609.557	522.798	1608.273	657.224	1609.183	543.751
101	1620.711	171.093	1619.366	128.001	1619.444	127.706
102	1626.839	325.895	1625.435	356.901	1625.885	366.108
103	1635.886	225.703	1635.086	186.606	1635.684	206.848
104	1646.220	8.304	1645.009	11.225	1645.981	10.302
105	1654.579	343.871	1652.991	279.045	1654.495	313.273
106	2988.739	44.366	2989.358	42.717	2989.230	43.585
107	3003.604	40.047	3003.753	41.080	3003.831	40.924
108	3058.791	34.225	3059.082	34.350	3058.853	35.181
109	3068.913	52.475	3069.050	52.426	3069.436	52.000

110	3117.305	26.236	3120.670	22.847	3123.505	19.887
111	3159.611	8.205	3159.356	8.063	3159.236	7.806
112	3174.062	9.603	3173.762	10.049	3173.821	10.598
113	3174.463	16.795	3174.156	17.269	3174.124	19.275
114	3177.262	3.247	3176.645	3.306	3176.049	3.799
115	3188.151	30.522	3187.836	30.615	3187.133	21.892
116	3190.543	19.000	3190.472	18.884	3190.044	20.203
117	3202.472	11.475	3202.019	12.848	3190.434	13.603
118	3204.776	5.301	3202.459	2.808	3200.166	16.985
119	3208.241	7.750	3207.579	8.478	3201.998	9.938
120	3229.058	7.931	3215.223	8.231	3206.441	11.566

Note on the computed harmonic vibrations. Animated GIF files showing selected computed harmonic modes together with their displacements vectors are attached to this manuscript as separate files. Animations comprise modes for the ground state (nitro form; wagging, scissoring and NO_2-group symmetric and asymmetric modes), and metastable state (*endo*-nitrito form; scissoring, N−O and N=O modes).

The Supporting Information is available free of charge and contains comprehensive synthesis and compound characterisation data, structure refinement details, crystal packing figures, supporting spectroscopic plots, cavity volume data, and additional computational data.

Accession codes:

CCDC 1975196-202 & 1975732-736 contain the supplementary crystallographic data for this paper. These data can be obtained free of charge via https://www.ccdc.cam.ac.uk/structures/, or by emailing data_request@ccdc.cam.ac.uk, or by contacting The Cambridge Crystallographic Data Centre, 12 Union Road, Cambridge CB2 1EZ, UK; fax: +44 1223 336033.
